# Machine Learning Approach for Predicting Drug-Like Molecules Targeting Calmodulin Pathway Proteins

**DOI:** 10.1021/acs.jcim.5c02111

**Published:** 2025-10-22

**Authors:** Maider Baltasar-Marchueta, Naia López, Sara Alicante, Iratxe Barbolla, Markel Garcia Ibarluzea, Rafael Ramis, Ane Miren Salomon, Arantza Muguruza-Montero, Eider Nuñez Viadero, Aritz Leonardo, Sonia Arrasate, Nuria Sotomayor, Matthew M Montemore, Alvaro Villarroel, Aitor Bergara, Esther Lete, Humberto González-Díaz

**Affiliations:** † Department of Organic and Inorganic Chemistry, 16402 University of the Basque Country UPV/EHU, Barrio Sarriena, s/n, 48940 Leioa, Bizkaia, Spain; ‡ Department of Chemical and Biomolecular Engineering, 5783 Tulane University, 6823 St. Charles Avenue, New Orleans, Louisiana 70118, United States; § Biofisika Institute, CSIC-UPV/EHU, Barrio Sarriena, s/n, 48940 Leioa, Bizkaia, Spain; ∥ 226245 Donostia International Physics Center, Manuel Lardizabal Ibilbidea, 4, 20018 Donostia, Gipuzkoa, Spain; ⊥ Department of Physics and EHU Quantum Center, 16402 University of the Basque Country UPV/EHU, Barrio Sarriena, s/n, 48940 Leioa, Bizkaia, Spain; # IKERBASQUE, Basque Foundation for Science, Euskadi Pl., 5, Abando, 48009 Bilbao, Bizkaia, Spain

## Abstract

Recently, numerous models have been developed to predict drug interactions with molecules. However, integrating diverse data sources and improving the accuracy of biological activity predictions remains a challenge. This work proposes a novel solution that addresses these limitations. Here, we have developed a machine learning model to predict the efficacy of different assays and drugs for diseases related to calmodulin. To achieve this, we have compiled a comprehensive data set including commercialized drugs and experimental compounds targeting CaM complexes. The IFPTML-XGB model achieved high predictive performance, with a test accuracy of 89.1% and a sensitivity of 89.0%, demonstrating its robustness for assay efficacy prediction. We have used the IFPTML modeling technique to identify key factors influencing these activities. We have also synthesized novel riluzole derivatives and have tested them both experimentally and computationally. Biological assays and molecular docking studies have been performed to provide a molecular-scale picture of the molecule–CaM interaction. To validate the model’s utility, we tested it on these derivatives. We have found that the model correctly predicts which derivatives were the most bioactive, indicating that this framework can be used to identify promising candidates for new drug formulations. This research not only improves our understanding of CaM-related diseases, but also provides an effective framework for developing new treatments based on predictive modeling.

## Graphical abstract

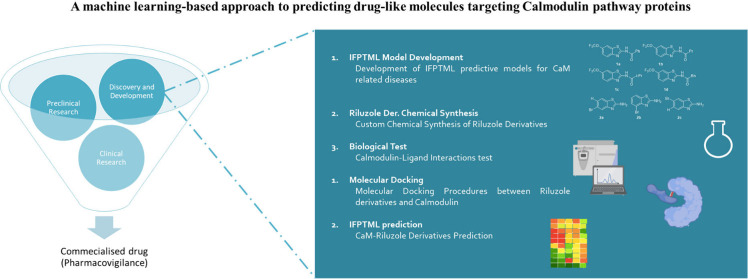

## 1 Introduction

While developing new drug formulations holds promise for improved treatments, the process is long and expensive.^1^ For this reason, improved efficiency and accuracy for predicting the efficacy of possible new drug formulations could save time and resources, and accelerate the improvement of health outcomes. To address this challenge, the development of improved computational tools, such as cheminformatics models, could enhance the effectiveness of medicinal chemistry.^2,3^

While computational tools have revolutionized drug development, many traditional chemoinformatic models struggle to handle the complexity and volume of big data in modern research.^4,5^ This limitation highlights the need for advanced approaches, such as machine learning (ML), which can provide more robust and accurate predictions.^6^ To address the need for models that can learn from large and heterogeneous data sets, our group developed the IFPTML methodology: information fusion (IF), perturbation theory (PT), and artificial intelligence and machine learning (AI/ML). Even though IFPTML models have effective across several medicinal chemistry applications.^7–10^ Yet, there remains a need for a clear demonstration of their effectiveness in a well-designed model drug discovery case study.

These computational methods can significantly impact the study of protein targets with critical biological roles.^9,11^ CaM, a key mediator in the calcium signaling pathway, is one such target of great interest due to its structural complexity and central role in cellular function.^12,13^ In fact, the influx of Ca^2+^ from the environment or release from internal stores causes a rapid and dramatic increase in cytoplasmic calcium concentration. In this context, CaM acts as an intermediate calcium sensor (Figure 1).^14^

**Figure 1:**
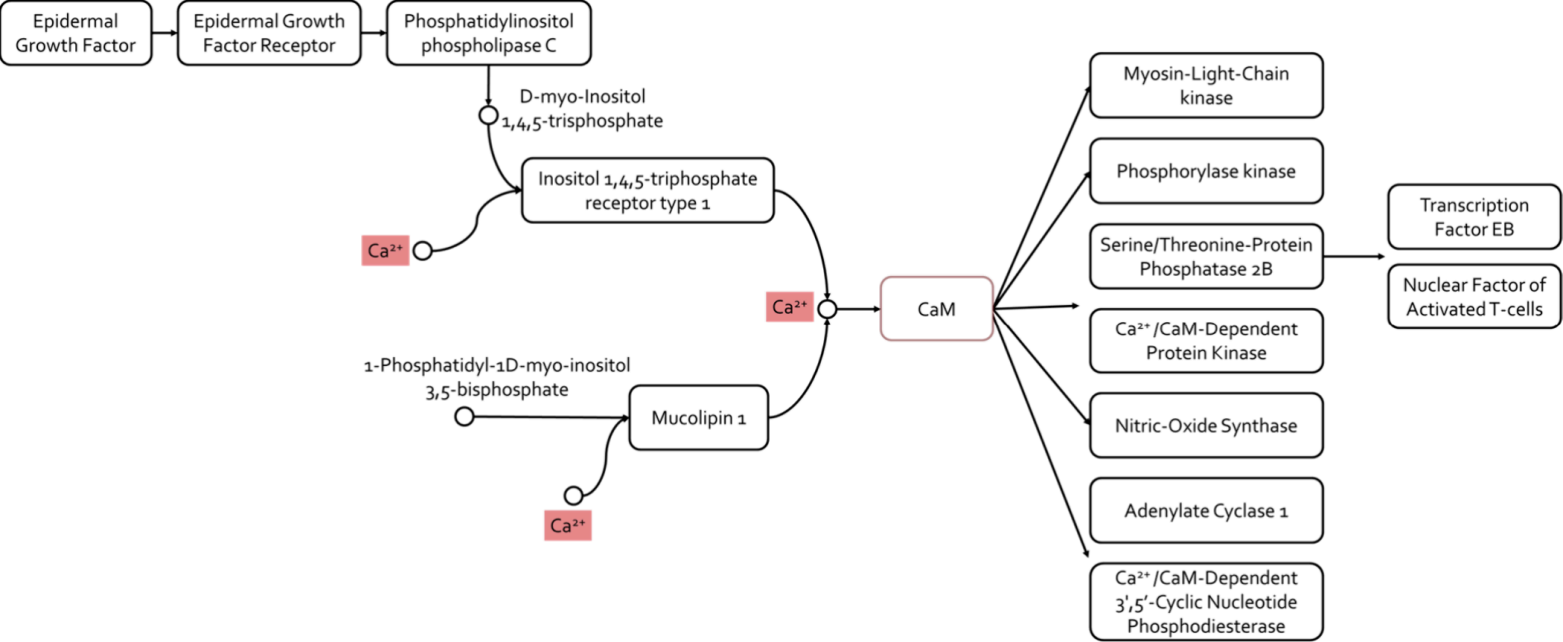
Calcium signaling pathway, focused on CaM-related proteins.^14^

CaM also plays a role in diseases with severe clinical implications. Most notably, CaM is associated with cardiopathies,^15–17^ calmodulinopathies, and neurodegenerative diseases.^18–21^

While our research group has previously developed a general ML-based model to study neurodegenerative diseases,^22^ here we develop and apply a targeted model focusing specifically on diseases related to CaM and related proteins in the calcium signaling pathway. In particular, the main objective of this work is to build a chemoinformatic model to predict the efficacy of different assays and drugs for diseases related to CaM. Our goal in developing IFPTML models is to predict *assay efficacy*, defined here as the likelihood that a compound will be active (i.e., *f*(*v_ij_*) = 1) under specific assay conditions, including particular protein targets, experimental concentrations, and assay types. Rather than evaluating assay quality per se, our focus lies in estimating compound performance across a diverse range of biological and experimental contexts. These models aim to guide the selection of promising compound–target–assay combinations, helping to prioritize candidates for experimental validation and reduce unnecessary screening efforts.

Furthermore, a case study was conducted specifically for riluzole derivatives (Figure 2). This involved chemical synthesis, biological testing, and docking calculations. Finally, the IFPTML model was applied to predictively evaluate the efficacy of these riluzole derivatives, demonstrating its utility in efficiently identifying promising candidate for future study.

**Figure 2:**
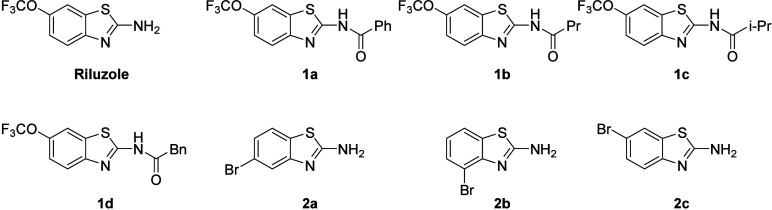
Proposed structures of riluzole and its derivatives.

## 2 Results and Discussion

### 2.1 IFPTML Models

In order to build the chemoinformatic model, information on various tests focusing on proteins related to CaM within the Ca^2+^ signaling pathway, as identified via the KEGG encyclopedia,^14,23–26^ were obtained. Drug and protein descriptors were either retrieved from ChEMBL,^23,25,26^ NCBI,^27,28^ or calculated using MARCH-INSIDE 2.0 (Markovian Chemicals In Silico Design)^29,30^ (see Table 1). Drug-related descriptors such as molecular weight, Lipinski’s Rule of Five (Ro5), and octanol/water partition coefficient (AlogP) were retrieved directly from the ChEMBL database. Additionally, assay-specific variables such as substrate and inhibitor concentrations were also obtained from ChEMBL assay metadata. On the other hand, more complex descriptors such as interatomic electronegativity, van der Waals forces, and contributions to AlogP between atoms were calculated using the MARCH-INSIDE. Likewise, using diverse tools provided by the National Centre for Biotechnology Information (NCBI)^31–34^ Web site, the FASTA sequence of the proteins tested in those ChEMBL trials were obtained. Protein descriptors were also computed based on amino acid electronegativity values extracted from the FASTA sequences of three functional domains. MARCH-INSIDE was also used to compute protein domain descriptors, including electronegativity at five different levels for each of the three biologically active domains of each target protein. Descriptors computed using MARCH-INSIDE involved propagation of physicochemical properties through atomic/molecular graphs, using up to five levels of atomic adjacency (Levels 0 to 5).

**Table 1:** Names, Meaning and Abbreviations of the Descriptors (D) and Variables (V) Used to Build the Cheminformatics Model

name (label)	descriptor or variable information
molecular weight (D_1_(drug))	the molecular weight of the drug
Lipinski’s rule of five (D_2_(drug_i_))	Lipinski’s rule of five for the drug
*n*-octanol/water partition coefficient (D_3_(drug_i_))	*A* log *P* value for the drug
electronegativity (D_001_(drug_i_)–D_035_(drug_i_))	avg electronegativity difference between each atom of the drug and the adjacent atoms^a^
van der Waals forces (D_036_(drug_i_)–D_070_(drug_i_))	avg of the van der Waals forces between each atom of the drug and the adjacent atoms^a^
contribution to the *n*-octanol/water partition coefficient (D_071_(drug_i_)–D_105_(drug_i_))	avg of *A* log *P* between each atom of the drug and the adjacent atoms
substrate concentration (V_1_)	concentration of the substrate used on the assay (μM)
inhibitor concentration (V_2_)	concentration of the inhibitor used on the assay (μM)
Electronegativity of first domain D_1_(prot_t_,dom_I_)–D_5_(prot_t_,dom_I_)	avg of first domain electronegativity for the five different levels^a^
electronegativity of second domain D_1_(prot_t_,dom_II_)–D_5_(prot_t_,dom_II_)	avg of second domain electronegativity for the five different levels^a^
Electronegativity of third domain D_1_(prot_t_,dom_III_)–D_5_(prot_t_,dom_III_)	avg of third domain electronegativity for the five different levels

a In this work, when adjacent atoms or different levels are indicated in a drug or protein descriptor, the following must be specified: this specific descriptor is calculated for some atoms (level 0) or also between these atoms and the adjacent atoms (level 1), these atoms + adjacent atoms + next-nearest neighbors (level 2), and so on. This sequence continues until 5 levels are reached in some calculated descriptors. These descriptors are calculated with the software MARCH-INSIDE 2.0 (*Markovian Chemicals In Silico Design*).^41,29,42,30^

These descriptors were selected based on their relevance to predicting interactions and their established use in previous studies.^35,36^ For instance, properties like molecular weight, Lipinski’s Rule of Five, and the octanol/water partition coefficient (*A* log *P*) have been widely used in cheminformatics due to their predictive power and the ease with which they can be calculated for new drug formulations.^37–39^ Additionally, electronegativity differences and van der Waals forces have proven to be valuable in describing molecular interactions in our previous work, making them ideal candidates for inclusion in this model.^40^

Next, the data set created in the Excel program was treated. Perturbation Theory Operators (PTOs) were estimated using Box-Jenkins Moving Average (MA) methods.^43^ These operators accounted for varying assay conditions, dividing them into assay-based and data-based boundary conditions. The objective function was also calculated for each of the assays the following way: since distinct assays used varying activity metrics (e.g., IC_50_, *K_i_*), desirability parameters and cut-offs were decided to standardize classifications. This way, drug activity values were transformed into Boolean variables 0 and 1 (objective function) to distinguish active (1) from inactive (0) compounds, ensuring consistency across assays. A reference function was then computed to estimate the probability of a compound being active under specific conditions. Finally, for predictive modeling, both linear and nonlinear classification techniques were employed.^44^ Further details on the algorithms are provided methods section. The data set used for model training and validation was released in a machine-readable CSV format, following the reproducibility guidelines of JCIM.^45^

The final data set included compounds, biological activity data, and various assay types. Notably, ChEMBL compounds included 1052 chemical entities, many of which are FDA-approved drugs such as Gefitinib and Tamoxifen, along with investigational compounds. Biological activities were tested across 13 different measures, including parameters like IC_50_ (nM), *K_i_* (nM), inhibition percentages, and potency (nM). The assays covered various target proteins, with key examples including Calmodulin (CaM), Myosin light chain kinase, and Epidermal growth factor receptor (EGFR) (101 targets). Furthermore, 11 diverse assay cell types were used, such as HEK293 (human embryonic kidney cells), CHO (Chinese hamster ovary cells), and RAW264.7 (mouse macrophage cell line). Assays covered diverse tissues and organisms, including *Homo sapiens*, *Mus musculus*, and *Plasmodium falciparum* (the malaria pathogen) (19 target organisms and 25 assay organisms). The total number of data points and different assays included in the final data set amounts to 3748. Table 2 summarizes the full list of assay conditions included in the data set.

**Table 2:** Summary of the Final Dataset Composition and Assay Conditions

	CHEMBL ID	*n*	examples
	compounds	1052	adenine, midostaurin, afimoxifene, imidazole, tivozanib, ...
	assay unique data points	3748	
c_0_	biological activity	13	residual activity (%); IC_50_ (nM); *K_i_* (nM), ...
c_1_	target name	101	CaM kinase II gamma; CaM kinase II delta; CaM kinase II beta; Myosin light chain kinase; ...
c_2_	assay cell type	11	MDCK; CHO; HEK293; CHO-K1; ...
c_3_	assay tissue name	9	brain; heart; Piriform cortex; testis; ...
c_4_	target organism	19	*Homo sapiens*; *Rattus norvegicus*; *Bos taurus*; *Plasmodium falciparum*; ...
c_5_	assay organism	25	*Sus scrofa*; *Cavia porcellus*; *Leishmania major*; *Cricetulus griseus*; ...
c_6_	target type	7	single protein; organism; protein family; ADMET, ...
c_7_	assay subcellular fraction	2	membrane; microsome
c_8_	buffer	7	8 mM MOPS, pH 7, 0.2 mM EDTA, 0.5 mM CaCl_2_; 40 mM HEPES, pH 7.4, 5 mM CaCl_2_; 40 mM HEPES, pH 7.4, 5 mM CaCl, 2.5 mM CaCl_2_, ...
c_9_	standard relation	3	equal (=); bigger (>); lower (<)
c_10_	assay type	4	binding (B); functional (F); ADMET (A); physicochemical (P)

To optimize the performance, a two-step feature selection process was implemented: variance thresholding and correlation-based filtering.^46,47^ A variance threshold of 0.01 was chosen to remove features with negligible variability across the data set, as these provide minimal discriminatory power.^48^ Moreover, features with very high pairwise correlations (|*r*| > 0.97) were considered redundant, based on prior studies indicating that multicollinearity beyond this threshold may impair model interpretability and stability (Supporting Information I, Figure S1).^49,50^ Consequently, a refined subset of 71 input features was retained (see Supporting Information II, Table S10).

Two data-splitting strategies were implemented to assess the robustness of the models and mitigate potential bias (Table 3). First, a random stratified split (80/20) was applied to preserve class balance while generating the training and testing partitions. In parallel, a cluster-based strategy was employed as a more structure-aware alternative. Specifically, K-Means clustering (*k* = 5) was applied to the standardized feature space, and an 80/20 split was performed within each cluster.^51,52^

**Table 3:** Dataset Composition by Activity Label and Train/Test Split

partition	inactive (*f*(*v_ij_*) = 0)	active (*f*(*v_ij_*) = 1)	total
training	1462	1535	2997
test	367	384	751
total	1829	1919	3748

For the hyperparameter optimization RandomizedSearchCV and GridSearchCV were performed.^44^ These methods were applied to fine-tune the model’s hyperparameters and improve its predictive accuracy. On the one hand, RandomizedSearchCV is a technique that performs a random search over a specified hyperparameter space, sampling a fixed number of parameter combinations. In the case of XGB, RandomizedSearchCV was used to search over a range of hyperparameters, including the number of estimators, learning rate, maximum depth, and other key parameters. Once the best hyperparameters were found through RandomizedSearchCV, GridSearchCV was used for fine-tuning. GridSearchCV searched through a specified grid of hyperparameters, evaluating every combination to find the best-performing model. In this case, GridSearchCV explored a refined set of hyperparameters around the best values obtained from the randomized search.

#### 2.1.1 IFPTML LDA Model

The proposed IFPTML-LDA classification model predicts whether a given compound will interact with a given protein. This model includes the reference function (*f*(*v_ij_*)_ref_), as well as the PT operators (PTOs) under a set of boundary conditions (Δ*D*_k_(drug_i_, c_j_), Δ*D*_k_(prot_t_, dom_m_, c_j_) and Δ*V*_k_(c_j_). The reference function estimates how likely a given case is to be successful compared to other cases with the same boundary conditions. Additionally, the PT operators quantify how much a particular case deviates from the average behavior of cases under the same conditions. First, we developed a model using linear discriminant analysis (LDA),^53,54^ which is highly interpretable and computationally efficient. Hyperparameter optimization^44^ for LDA was conducted using random search (testing 15 parameter combinations), resulting in the least-squares solver and no shrinkage as the best parameters. A grid search was used to optimize the hyperparameters of the IFPTML-LDA model. To assess the influence of the data partitioning strategy, the model was evaluated under both a random 80/20 split and a K-Means-based split. The random partition yielded a test accuracy of 78.53%, while the K-Means-based split achieved 79.49% (Table 4). Although the cluster-based split produced a marginal increase in performance (approximately 1%), the difference was not substantial enough to indicate a change in the model’s generalization behavior. For this reason, and to ensure comparability with previous studies and standard practice in the field, the random split was retained as the primary strategy for subsequent analyses.

**Table 4:** Classification Accuracy and Statistical Parameters for the IFPTML-LDA Model

					predicted classification		
data split	data set	observed classification	stat. param^a^	pred. stats	*n_j_*	*f*(*v_ij_*)_pred_ = 0	*f*(*v_ij_*)_pred_ = 1
random	training (80%)	*f*(*v_ij_*)_obs_ = 0	Sp (%)	70.45	1462	1030	432
		*f*(*v_ij_*)_obs_ = 1	Sn (%)	89.71	1536	158	1378
		total	Ac (%)	80.32	2998	1188	1810
	test (20%)	*f*(*v_ij_*)_obs_ = 0	Sp (%)	66.21	367	243	124
		*f*(*v_ij_*)_obs_ = 1	Sn (%)	90.34	383	37	346
		total	Ac (%)	78.53	750	280	470
K-means	training (80%)	*f*(*v_ij_*)_obs_ = 0	Sp (%)	70.38	1462	1029	433
		*f*(*v_ij_*)_obs_ = 1	Sn (%)	89.44	1535	162	1373
		total	Ac (%)	80.14	2997	1191	1806
	test (20%)	*f*(*v_ij_*)_obs_ = 0	Sp (%)	68.39	367	251	116
		*f*(*v_ij_*)_obs_ = 1	Sn (%)	90.10	384	38	346
		total	Ac (%)	79.49	751	289	462

a Statistical parameters of the model: Sp: specificity; Sn: sensitivity; Ac: accuracy; *n*: the number of cases used to train the model.

The model also demonstrated high predictive accuracy as measured by the area under the receiver operating characteristics curve (AUC), with a training AUC of 0.886 and a test AUC of 0.866. In addition, the model achieved a Matthews Correlation Coefficient (MCC) of 0.615 and Cohen’s kappa of 0.604 on the training set, and MCC of 0.584 and kappa of 0.568 on the test set. These results suggest that the model performs well, with a high recall (sensitivity) but slightly lower specificity, indicating its strength in identifying positive cases. To provide a more comprehensive evaluation of the model’s performance, additional metrics were calculated. For the training set, the LDA model achieved a precision of 0.813, recall of 0.803, and F1-score of 0.801. On the test set, the precision was 0.801, recall 0.785, and F1-score 0.782. The confusion matrices for LDA are presented in Figure 3.

**Figure 3:**
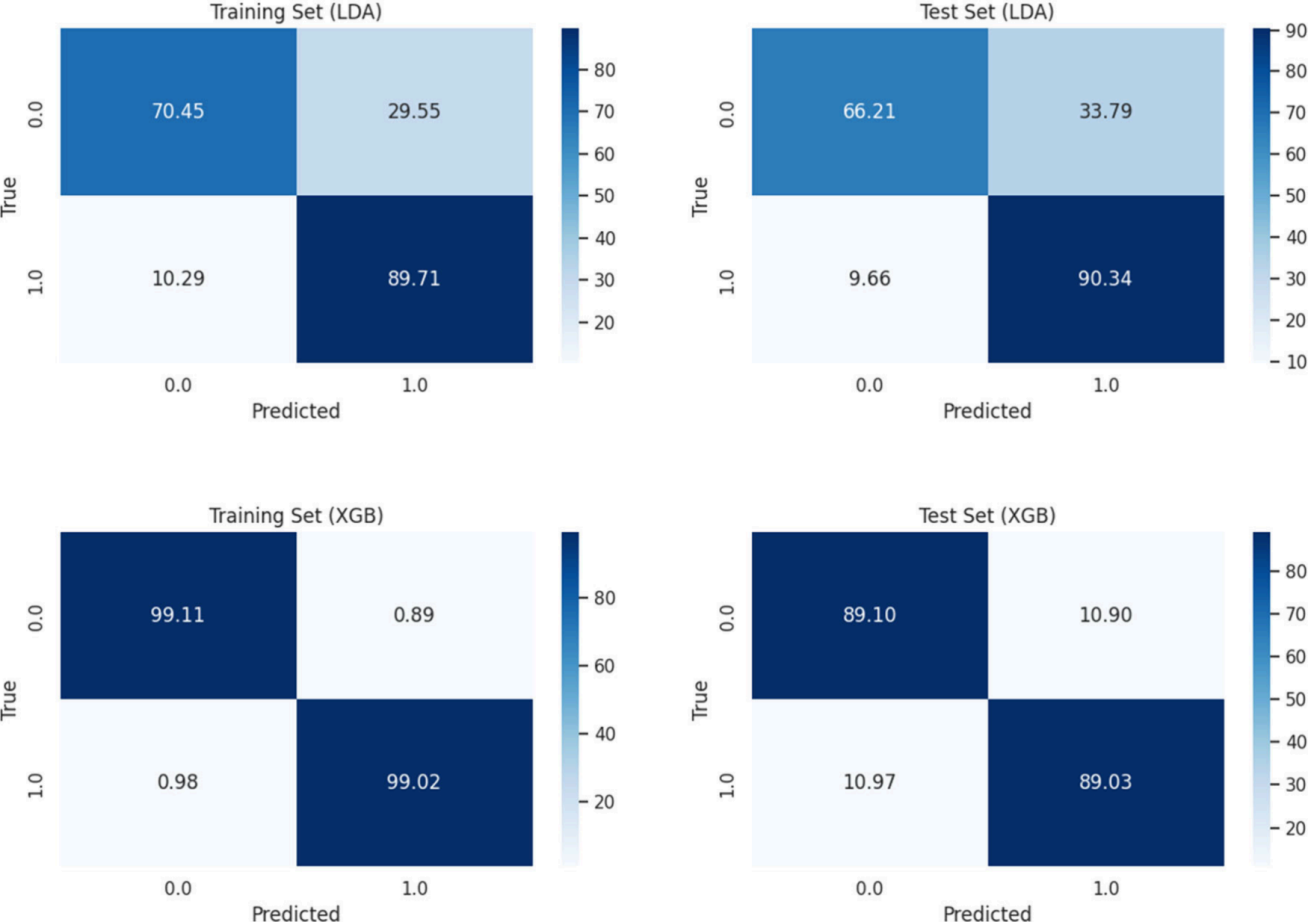
Confusion matrices for model performance evaluation. LDA and XGB models; Results in %.

Furthermore, the importance of individual features was evaluated for the LDA model, with the top 20 most influential features visualized in Figure 4.

**Figure 4:**
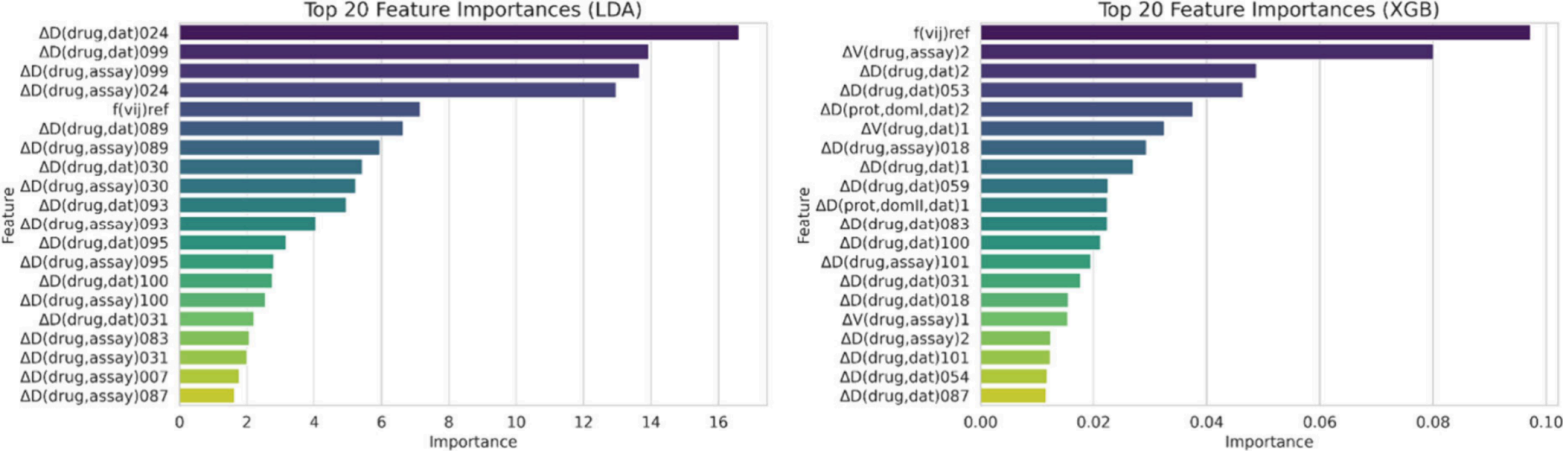
Top 20 feature importances for LDA and XGB models.

The top 20 most relevant features primarily focus on electronegativity and lipophilicity (AlogP)^55^ of different atomic groups, particularly heteroatoms, halogen atoms, labile hydrogen atoms, and unsaturated carbons at various levels. The model also includes a reference function related to the *a priori* probability of a compound interacting with proteins in specific conditions. These descriptors suggest that the electronic properties and solubility-related features of the drugs play a crucial role in distinguishing active from inactive compounds in the LDA model. The top features in the LDA model are predominantly small-molecule descriptors, with minimal representation of protein-derived variables. Although this bias contributes to good predictive performance within the chemical space represented in the data set, it has important implications for model generalizability and interpretability. The model may implicitly assume that similar compounds behave similarly across different protein targets. This could limit extrapolation to protein families underrepresented during training. However, in this particular study, the targets considered belong to a biologically coherent group, i.e., proteins associated with the CaM in the calcium signaling pathway, such as calmodulin, CaM-dependent kinases (e.g., CAMK I, II, and III), and related regulatory proteins. Within such a functionally and structurally conserved protein subset, the lower contribution of protein-derived descriptors is expected, as limited variability among targets reduces their discriminative power relative to compound features. Thus, future extensions incorporating richer protein data could help balance the contributions of both molecular and protein variables and improve transferability. Table S1 in Supporting Information I, summarizes the information about these top 20 features.

One of the key advantages of using a LDA model is its interpretability, allowing us to observe how specific features influence the prediction as discussed above. Additionally, its high sensitivity and balanced performance make it a useful tool for initial screening and understanding fundamental structure–activity relationships. Nevertheless, LDA’s linear nature may limit its ability to capture complex relationships within the data.

#### 2.1.2 IFPTML Nonlinear Models

To improve the IFPTML-LDA model’s sensitivity and accuracy, various nonlinear models were developed. The analysis involved several nonlinear models, including random forest (RF),^56^ support vector machine (SVM),^57^ decision tree (DT),^58^ K-nearest neighbors (KNN),^59^ gradient boosting (GB)^60^ and XGBoost (XGB).^61^ In Supporting Information Section 1.2, further information about the different models is summarized. To evaluate and select the best-performing model, we applied 10-fold cross-validation.

ROC curves were generated to evaluate the performance of these classification models.^62^ They plot the true positive rate (sensitivity) against the false positive rate (1 - specificity) at various classification thresholds (see Figure 5). The AUC quantifies the overall performance of the model, with values closer to 1 indicating better discriminative ability. Among the models, XGB and RF exhibited the highest AUC scores, with AUC test scores of 0.95 for both.

**Figure 5:**
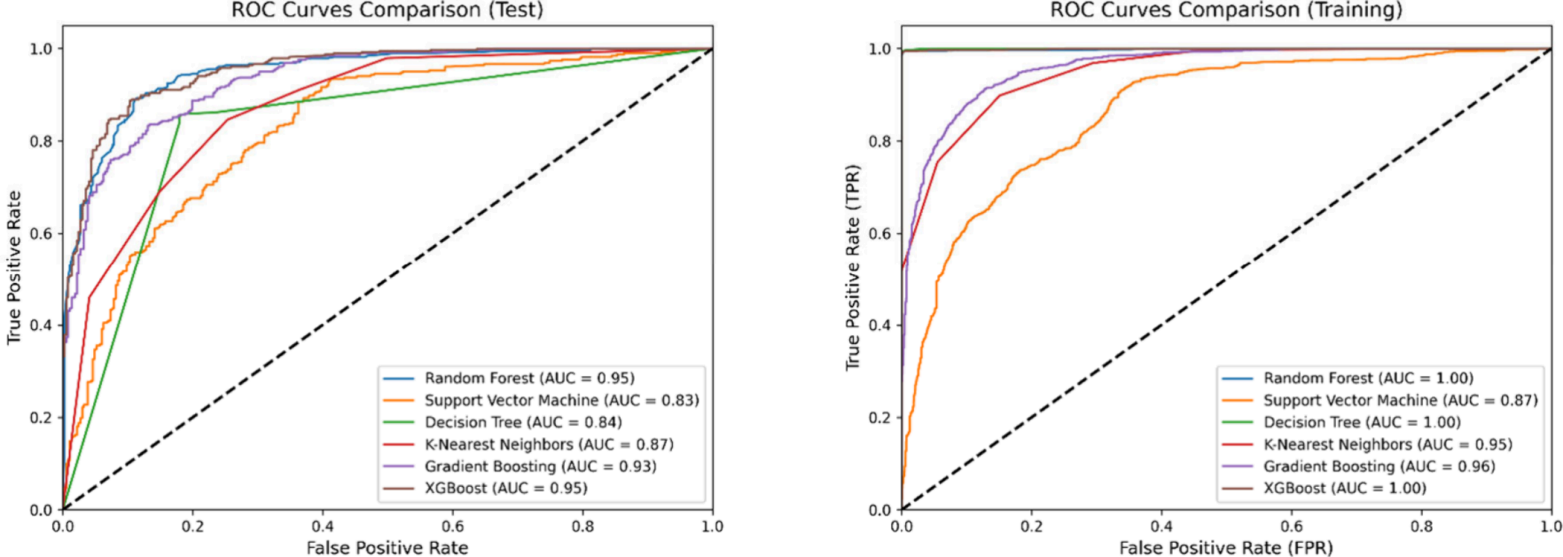
ROC curves for training and test for nonlinear models.

The results showed that XGB achieved the highest mean accuracy (0.87) and ROC AUC (0.94) during cross-validation, making it the top candidate for final testing. After selecting XGB we retrained it on the full training data set and evaluated its performance on the test set. The final accuracy (0.89) and ROC AUC (0.95) were consistent with the cross-validation results, indicating that the model generalized well without significant overfitting. These findings suggest that XGB is a robust and reliable model for our classification task.

Subsequently, hyperparameter optimization^44^ for XGB was conducted using random search, testing 45 parameter combinations. This process identified a subsample rate of 1.0, 170 estimators, a maximum tree depth of 10, and a learning rate of 0.267 as key parameters. A grid search further refined these values, increasing the maximum depth to 12 and adjusting the number of estimators to 160. To evaluate the impact of the data-splitting strategy, both random 80/20 partitioning and K-Means–based splitting were applied. The random split achieved the highest performance, with a test accuracy of 89.07%, compared to 88.41% under the K-Means split. In addition, the random split showed slightly better balance between specificity and sensitivity on the test set and preserved the strong generalization capacity of the model. Because this strategy yielded the most favorable and stable results, it was selected as the primary reference for subsequent analyses. The complete metrics for both approaches are shown in Table 5.

**Table 5:** Classification Accuracy and Statistical Parameters for the IFPTML-XGB Model

					predicted classification		
data split	data set	observed classification	stat. param^a^	pred. stats.	*n_j_*	*f*(*v_ij_*)_pred_ = 0	*f*(*v_ij_*)_pred_ = 1
random	training (80%)	*f*(*v_ij_*)_obs_ = 0	Sp (%)	99.11	1462	1449	13
		*f*(*v_ij_*)_obs_ = 1	Sn (%)	99.02	1536	15	1521
		total	Ac (%)	99.07	2998	1464	1534
	test (20%)	*f*(*v_ij_*)_obs_ = 0	Sp (%)	89.10	367	327	40
		*f*(*v_ij_*)_obs_ = 1	Sn (%)	89.03	383	42	341
		total	Ac (%)	89.07	750	369	381
K-means	training (80%)	*f*(*v_ij_*)_obs_ = 0	Sp (%)	94.19	1462	1377	85
		*f*(*v_ij_*)_obs_ = 1	Sn (%)	95.77	1535	65	1470
		total	Ac (%)	95.00	2997	1442	1555
	test (20%)	*f*(*v_ij_*)_obs_ = 0	Sp (%)	88.01	367	323	44
		*f*(*v_ij_*)_obs_ = 1	Sn (%)	88.80	384	43	341
		total	Ac (%)	88.41	751	366	385

a Statistical parameters of the model: Sp: specificity; Sn: sensitivity; Ac: accuracy; *n*: the number of cases used to train the model.

The model also demonstrated outstanding AUC values beyond the LDA model, with a training AUC of 0.999 and a test AUC of 0.953. These results suggest that the model is highly accurate and balanced, with a high recall (sensitivity) and specificity, indicating its strength in identifying all cases. In addition, the model achieved a MCC of 0.981 and Cohen’s kappa of 0.981 on the training set, and MCC of 0.781 and kappa of 0.781 on the test set. The confusion matrices for XGB are presented in Figure 3. Additionally, we computed the precision, recall, and F1-score for a more thorough assessment. The XGB model achieved a precision of 0.895, recall 0.890, and F1-score 0.893 on the test set, highlighting its robust classification performance even in the presence of false positives (10.90%) and false negatives (10.97%). Still, it is worth noting that the performance on the training set was notably higher (accuracy: 99.07%, AUC: 0.999) than on the test set (accuracy: 89.07%, AUC: 0.953), suggesting a moderate degree of overfitting. This is expected in complex models trained on high-dimensional feature spaces, despite careful regularization and feature selection. Nevertheless, the test metrics remain robust and well-balanced, indicating good generalization performance.

Additionally, the importance of individual features was evaluated for the XGB model, with the top 20 most influential features visualized in Figure 4 and Supporting Information I, Table S4. The XGB model’s top features highlight the reference function as most significant, with PTOs for drug and protein properties also playing key roles. The most influential descriptors cover a broader range of physicochemical and interaction-based properties. Key features include inhibitor and substrate concentration, molecular weight, van der Waals forces, electronegativity (of both drugs and protein domains), and AlogP values at different levels. Additionally, Lipinski’s rule of five appears multiple times, emphasizing the importance of drug-likeness in predicting activity. Unlike LDA, XGB effectively leverages protein-related features, which likely enhance its predictive power by considering both ligand and target properties. However, the overall imbalance in feature importance remains: the majority of top descriptors are still small-molecule-related. This suggests that while XGB better integrates protein-level information, the model may still be predominantly driven by ligand properties. As a result, the performance of the model for diverse protein targets could be limited.

XGB achieves high predictive accuracy via its nonlinearity. Furthermore, unlike LDA, XGB integrates both ligand and protein information as important features (top 20), making it more robust in identifying subtle structure–activity patterns. Due to its high accuracy, we chose to apply the XGB model to screen for promising riluzole derivatives to target CaM-related disorders.

### 2.2 Case Study of Riluzole Derivatives

Once the model was developed, a case study was carried out to test whether the model could be useful for predictions of drug activity in advanced. Especially, the goal was to test whether the model could be useful for predicting the activity of new riluzole derivatives. Synthesis, biological assays, and docking studies were performed to test these new compounds against CaM. Additionally, an IFPTML predictive study was performed to predict interactions between these new riluzole derivatives and all proteins in the Ca^2+^ signaling pathway related to CaM.

#### 2.2.1 Biological Assay of Riluzole Derivatives

After obtaining the riluzole derivatives in moderate yields (Supporting Information I, Section 2), the biological activity assay^22^ of these molecules was carried out. YC-Nano15 is a genetically encoded Ca^2+^ indicator based on Förster resonance energy transfer (FRET), designed to monitor intracellular calcium dynamics.^63^ Bearing that in mind, the FRET index for the original YC-Nano15 was determined in the presence and absence of Ca^2+^. Upon addition of 24 nM free Ca^2+^, the index increased from 2.7 ± 0.1 to 27.0 ± 0.1, with an EC_50_ of 2.1 ± 0.1 nM. The effect on the FRET amplitude of riluzole and related compounds (at a final concentration of 100 μM) was then measured. Riluzole and drugs **1d** and **2b** produced no significant effect. In contrast, compounds **1b** and **2c** produced a small inhibition and compounds **1a**, **1c**, and **2a** produced a more pronounced change by decreasing (**1c** and **2a**) or increasing (**1a**) the FRET index.

Next, we characterized the SK4 biosensor in the presence and absence of riluzole, as previously described in recent works.^22^ The SK4 biosensor exhibited a distinctive response to Ca^2+^, increasing the FRET index from 2.0 ± 0.5 to 8.7 ± 0.1. This response was altered by the presence of riluzole. The Ca^2+^ response displayed two discernible phases: Phase I at low Ca^2+^ concentrations (0–150 nM) and Phase II at higher concentrations (Figure 7). Notably, riluzole had a pronounced impact on Phase II, with EC_50_ values of 302.4 ± 22.3 nM and 221.3 ± 31.1 nM in the absence and presence of the drug, respectively (Figure 6).^64^

1 $$f=y_{0}+a\times \frac{x^{b}}{c^{b}+x^{b}}+d\times \frac{x^{e}}{g^{e}+x^{e}}$$

where *f* represents the response; *y*_0_ is the baseline response; *a* and *d* are the maximum effects of the two binding sites; *b* and *e* are the Hill coefficients; *x* is the Ca^2+^ concentration; and *c* and *g* are the EC_50_ values for each binding site.

**Figure 6:**
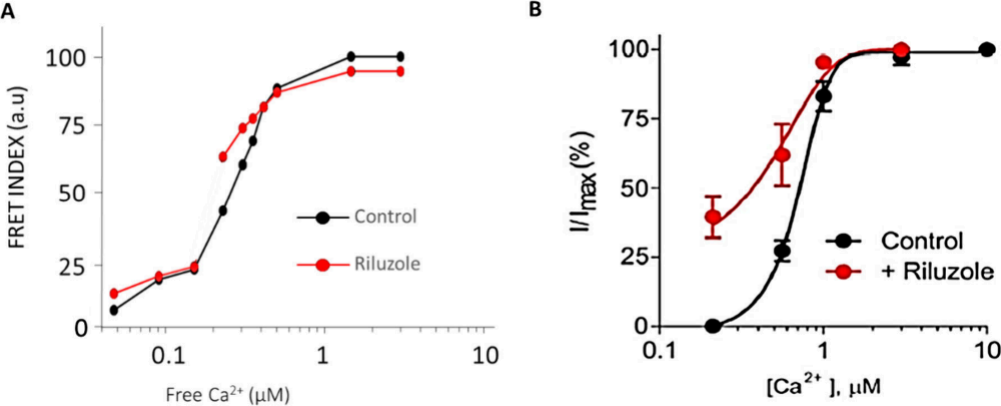
Ca^2+^ titration in the presence (red) and the absence (black) of riluzole (100 μM). (A) Titration using the SK4 biosensor. A two-binding site Hill eq (eq 1) was used to fit to the data. The EC_50_ values were 55.4 ± 23.8 (Phase I) and 302.4 ± 22.3 (Phase II) in the absence of riluzole and 24.7 ± 25.8 (Phase I) and 221.3 ± 31.1 (Phase II) in the presence of riluzole. Free Ca^2+^ concentration was estimated using Fura-27. (B) Calcium-activation curves for SK2 derived from inside-out patch-clamp experiments taken from ref 64.

To better understand how riluzole analogues affect calcium sensitivity and channel function, we next performed Ca^2+^ titrations using the SK4 biosensor in the presence of riluzole analogues. Unlike riluzole, none of the drugs significantly altered Ca^2+^ sensitivity. Instead, they caused a variable reduction of the amplitude. All compounds inhibited the second phase, with compound **2a** inducing the most pronounced reduction (74%) (Figure 7). However, the effects on the first phase vary depending on the compound: compound **2b** did not produce significant effects, while compounds **1a**, **1b**, **1c**, **1d** and **2c** produced an inhibition of the amplitude, whereas compound **2a** caused an increase (Table 6). Additional information is provided in Supporting Information I, Section 3.

**Figure 7:**
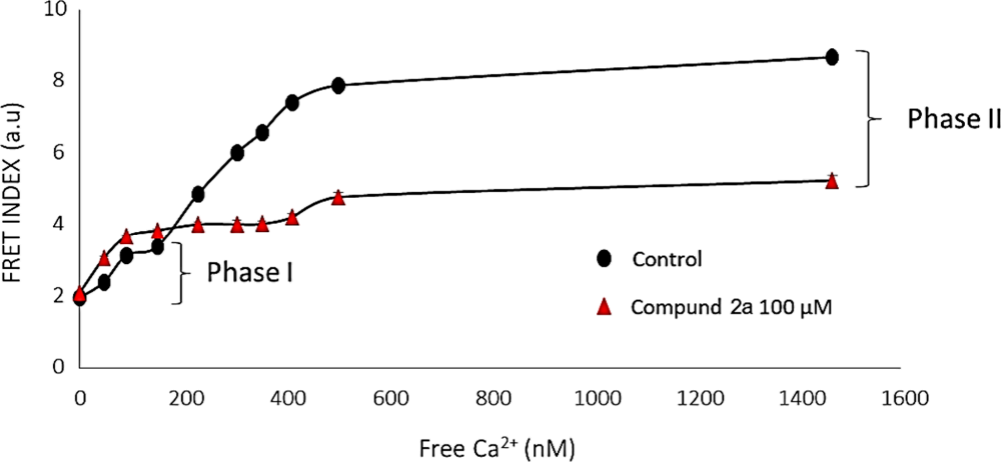
Ca^2+^ titration in the presence (red) and absence (black) of drug **2a**. Equation 1 was used to fit the curve to the data.

**Table 6:** Relative Amplitude in the Presence of the Indicated Drugs at 100 μM^a^

compound	total inhibition	Phase I inhibition	Phase II inhibition
riluzole	7.9 ± 2.3	–2.3 ± 2.3	10.7 ± 3.3
**1a**	26.0 ± 2.1	20.7 ± 3.6	27.5 ± 3.4
**1b**	26.7 ± 1.8	6.3 ± 1.2	32.2 ± 2.7
**1c**	18.6 ± 2.3	15.7 ± 10.7	19.3 ± 5.0
**1d**	26.7 ± 1.8	3.3 ± 6.5	32.8 ± 2.9
**2a**	53.3 ± 2.4	–21.9 ± 1.7	73.7 ± 3.4
**2b**	28.1 ± 1.8	0.8 ± 3.1	29.1 ± 2.5
**2c**	4.7 ± 2.4	6.1 ± 0.4	4.4 ± 1.1

a Mean ± standard error of the mean (SEM) (*n* ≥ 3). Total inhibition is the relative reduction of Phase I and Phase II amplitudes combined.

#### 2.2.2 Molecular Docking Study

To elucidate the binding mechanism of the analyzed compounds, we performed docking calculations for each compound at the interface between CaM and the S_45_A helix of the SK4 channel. Figure 8A shows the most stable conformation predicted by docking for riluzole, which we previously showed^65^ and is similar to previously reported structures.^64^ This conformation is characterized by the riluzole −OCF_3_ group pointing toward the hydrophobic pocket between CaM and SK4, with its −NH_2_ group forming a hydrogen bond with E54 at the opening of the pocket. Notably, the IFPTML-XGB model identified electronegativity-related features for labile hydrogens and halogens as key predictors, aligning with the observed importance of hydrogen bonding and the electronegative nature of the trifluoromethoxy (−OCF_3_) group in riluzole’s interaction with CaM.

**Figure 8:**
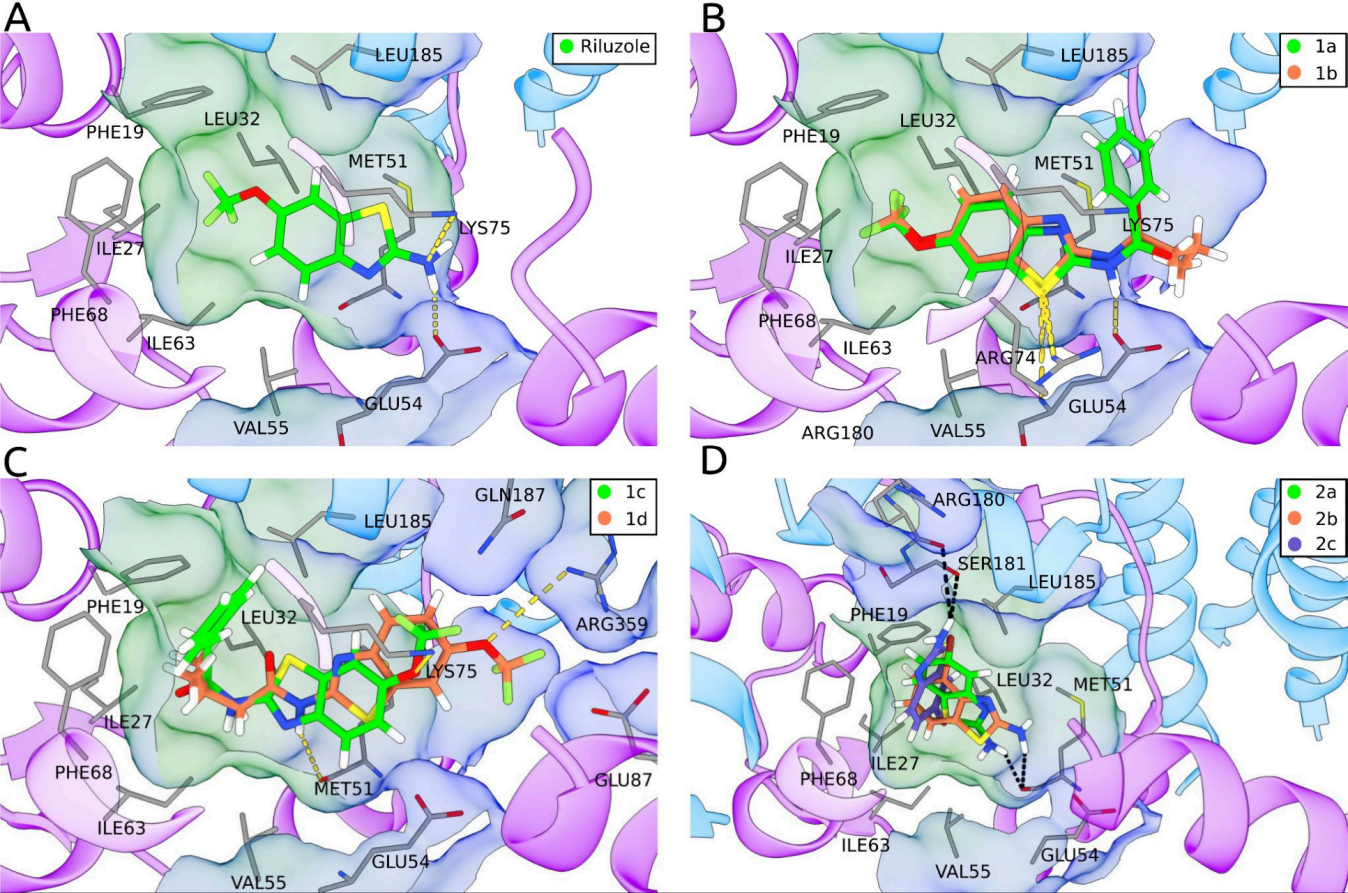
Docking results of the reported compounds at the interface between CaM and the S_45_A helix of the SK4 channel. Yellow lines indicate hydrogen bonds. The binding pocket surface is represented with a hydrophobicity-based coloring scheme, with green indicating hydrophobic regions, and blue indicating hydrophilic regions. (A) Best binding pose of riluzole determined through docking. (B) Conformations of ligands **1a** and **1b**, with equal orientation to riluzole, with their -OCF_3_ group pointing toward the hydrophobic pocket and their substituent groups oriented outward, while forming a hydrophobic interaction with E54. (C) Conformations of ligands **1c** and **1d**, with an orientation opposite to riluzole’s, with their hydrophobic substituents oriented toward the inside of the pocket and the −OCF_3_ group pointing toward the entrance of the pocket. (D) Conformations of ligands **2a**, **2b**, and **2c**, which lie completely inside the hydrophobic pocket, and do not interact with residues in the opening of the pocket.

The docked conformations of the ligands vary significantly. Ligands **1a** and **1b** resemble riluzole, with their −OCF_3_ group targeting the hydrophobic pocket, whereas **1c** and **1d** exhibit reversed orientations. Ligands **2a**–**2c**, lacking the −OCF_3_ group, position deeper in the pocket. To validate these conformations, we conducted both Binding Pose Metadynamics (BPMD) and extensive 100 ns molecular dynamics simulations, which confirmed the stability of all docked poses. MM-PBSA binding energy calculations performed on these 100 ns trajectories showed good correlation with experimental inhibition data. For a detailed explanation and validation, see Supporting Information I, Section 4.

#### 2.2.3 IFPTML Prediction and CaM Protein Pathway Selectivity

Riluzole derivatives’ activity was tested against CaM in a biological assay, but given CaM’s role in the broader Ca^2+^ signaling pathway, their binding affinity to other related proteins was also assessed using the IFPTML-XGB model. This allows for clearer insights into which features drive binding affinity, making it particularly useful for understanding structure–activity relationships. We developed several graphs to visually interpret the results obtained in the predictions (Figure 9). On the one hand, we compared the success probabilities of different compounds, including riluzole, its derivatives (**1a**–**2c**), and four other commercial drugs (clozapine, loperamide, midostaurin, and tivozanib). Notably, derivative **2c** exhibited the highest success probability, closely followed by group 1. Also, in a second bar plot, success rates were analyzed by assay type, revealing that the binding assay yielded the best results. This is particularly encouraging, as our biological tests between the derivatives and CaM are based on binding assays.

**Figure 9:**
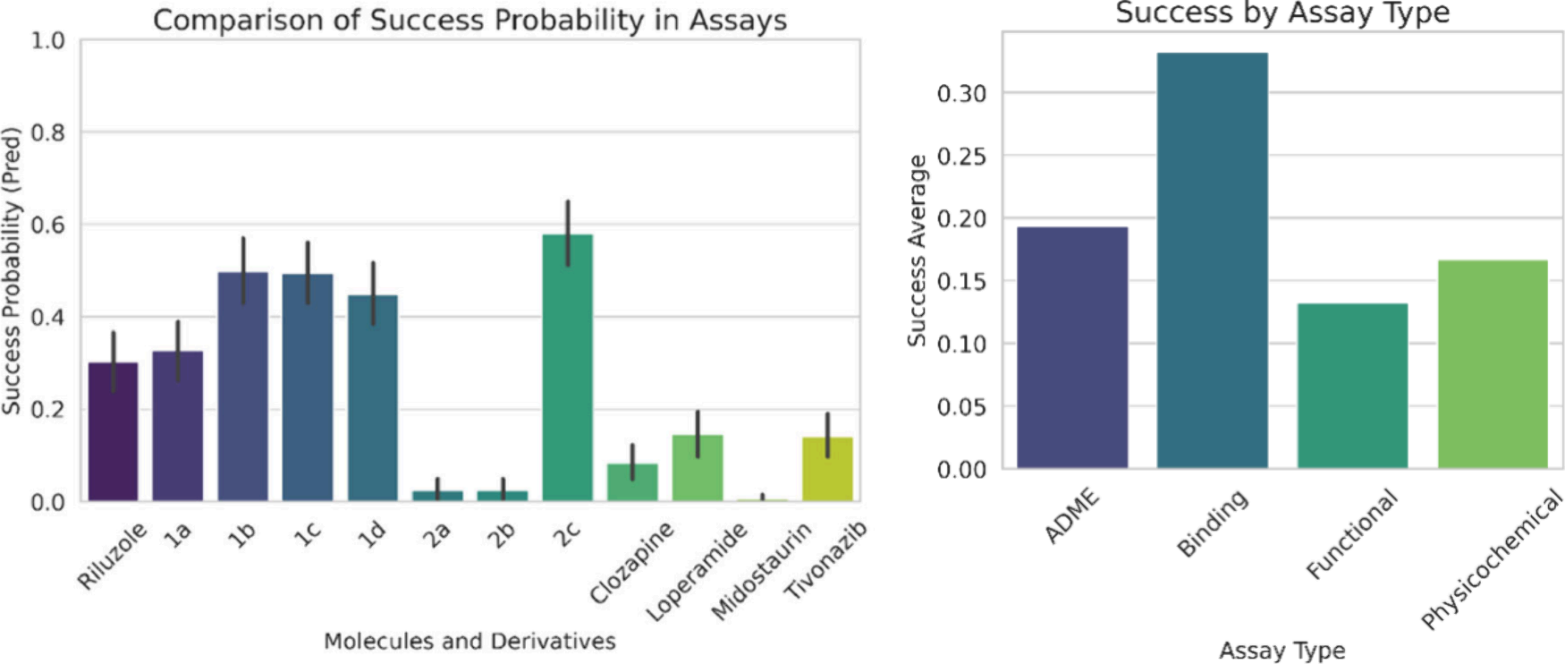
Results of the predictions graphed by success probability in assays and success by assay type.

Additionally, a heatmap was used to compare the activity of riluzole derivatives against CaM-related proteins (Figure 10). Proteins are listed on the left, whereas riluzole derivatives are shown on the bottom, with each column representing the IFPTML prediction outcomes. The values indicate the average relative outcome in binding activity (target: *Homo sapiens* cases) compared to riluzole (Δ*f*(*v_ij_*)_calc_). Warmer colors (red) denote weaker activity, while cooler colors (green) indicate stronger activity. Intensity reflects the significance of the interaction.

**Figure 10:**
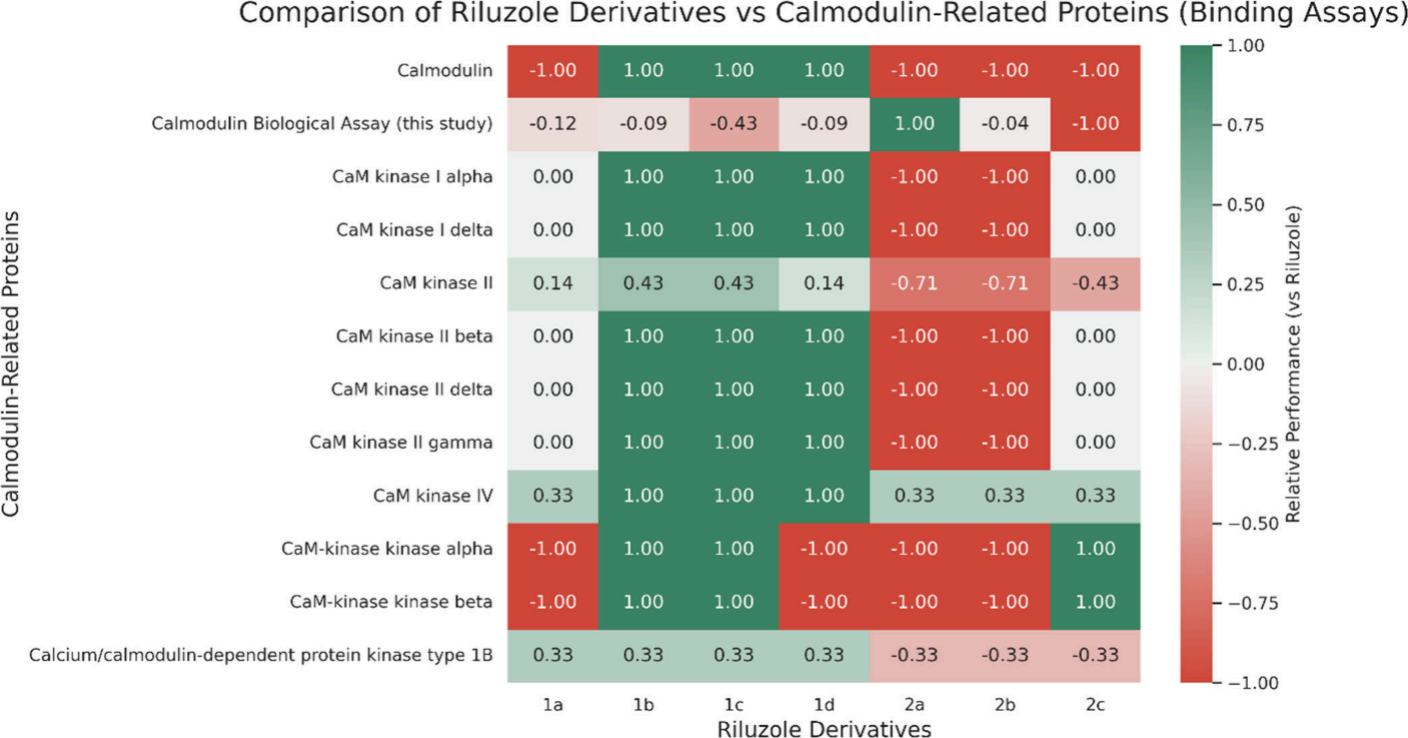
Different riluzole derivative’s prediction against proteins related to CaM, for preclinical assays that were performed in *Homo sapiens* genes.

The heatmap shows that compounds from family 1 generally have better binding affinity than group 2. Compounds **1b** and **1c** showed the highest binding affinity. However, a discrepancy arises when comparing these predictions to the experimental results. For instance, compound **2a** showed the strongest inhibition in the biological assay (Δ*f*(*v_ij_*)_calc_ = 1.00; 74% reduction in Phase II amplitude), yet in the heatmap it is predicted to have a lower binding affinity relative to riluzole (Δ*f*(*v_ij_*)_calc_ = −1.00). Conversely, compounds **1b** and **1c**, which demonstrated only moderate inhibition *in vitro*, are predicted to have enhanced activity. This apparent inconsistency comes from the fact that the IFPTML model was not trained on the same specific experimental conditions of our biosensor system described.^22^ Our IFPTML model predicts assay efficacy, the likelihood that a compound will be active (i.e., *f*(*v_ij_*) = 1) under specific assay conditions (not necessarily the same as the biosensor system). Thus, the inconsistency in the predicted results is retrieved from this. Also, the values gathered in the heatmap come from the average of the results of the relative value comparing to riluzole, in binding assays, in *Homo sapiens* lines. In Table 7 some of the specific cases are illustrated in which some of them are better and some of them are not.

**Table 7:** Specific Cases of the Data Used to Develop the Heatmap^a^

der.	*c*_01_	*c*_04_	*c*_05_	*c*_06_	*c*_08_	*c*_10_	pred.
riluz.	CaMKII	*Homo sapiens*	*Homo sapiens*	PROTEIN FAMILY	0	B	0
**1a**	CaMKI	*Homo sapiens*	*Homo sapiens*	SINGLE PROTEIN	0	B	0
**1b**	CaM	*Homo sapiens*	*Homo sapiens*	SINGLE PROTEIN	0	B	1
**1c**	CaM	*Homo sapiens*	*Homo sapiens*	SINGLE PROTEIN	0	B	1
**1d**	CaMKII	*Homo sapiens*	*Homo sapiens*	PROTEIN FAMILY	0	B	1
**2a**	CaM	*Homo sapiens*	*Homo sapiens*	SINGLE PROTEIN	0	B	0
**2b**	CaMKI	*Homo sapiens*	*Homo sapiens*	SINGLE PROTEIN	0	B	0
**2c**	CaMKI	*Homo sapiens*	*Homo sapiens*	SINGLE PROTEIN	Buffer: 8 mM MOPS, pH 7, 0.2 mM EDTA, 0.5 mM CaCl_2_	B	1

a der: derivatives; pred: predicted value by XGBoost model under certain boundary conditions; *c*_01_ = target name, *c*_04_ = target organism, *c*_05_ = assay organism, *c*_06_ = target type, *c*_08_ = buffer, *c*_09_ = std relation, *c*_10_ = assay type (B: binding).

Thus, the IFPTML model should be understood as a tool that guides compound selection. Importantly, its predictions need to be complemented with experimental validation under the specific assay conditions of interest. The model still provides valuable insights to focus experimental efforts more efficiently and reduce the risk of overlooking promising candidates.^66^

## 3 Conclusions

This research highlights the successful development of chemoinformatic models, particularly LDA and XGB models. The LDA model achieved high specificity, sensitivity, and overall accuracy while maintaining simplicity and interpretability, whereas the XGB model showed exceptional training and test accuracy. Additionally, the synthesis and biological assays of novel riluzole derivatives demonstrated their interactions with CaM, supported by docking studies. Last but not least, the predictive model identified compounds with strong binding affinities, indicating their potential for further development.

Moreover, as for the riluzole derivatives, **2a** showed the largest inhibition in the biological assays and deep pocket binding in docking. However, group 1 also showed promising results since its results from biological assays, docking studies and ML predictions, more precisely, **1b**, **1c**, and **1d**. In fact, they showed strong binding affinities to CaM in ML predictions, aligning with their moderate inhibitory effects observed in biological assays and their riluzole-like binding orientations in docking studies. The differences between the compounds can be attributed to the structural variations between the two groups: group 1 compounds are N-acyl and N-benzoyl riluzole derivatives, retaining the −OCF_3_ group crucial for interactions at the pocket entrance and allowing them show similar results as riluzole; whereas group 2 compounds are brominated benzothiazole derivatives lacking the −OCF_3_ group, allowing deeper binding but differing interactions with CaM in ML predictions.

Overall, this work advances the understanding of CaM-related diseases by establishing a novel drug discovery framework that integrates experimental and computational methods. Our ML models not only predict binding affinities with CaM but also assess interactions with other relevant proteins, enabling more comprehensive screening. The validation with new compounds demonstrates the framework’s potential for efficiently identifying promising drug candidates, paving the way for therapeutic strategies targeting calmodulinopathies and other CaM-associated conditions.

## 4 Materials and Methods

In this project five parts were involved (Figure 11). First, a predictive model was developed. The main aim of this model is to predict successful drugs for diseases related to CaM. The model should take into account the drug used on preclinical assays and the protein involved on the calcium signaling pathway related to CaM. Moreover, considering this model and using riluzole as benchmark molecule, 7 derivatives were proposed. Each of these distinct molecules were tested to see if they were successful against CaM or not. This testing included the synthesis of the molecules, the biological test against CaM, as well as molecular docking and the prediction with the predictive model.

**Figure 11:**
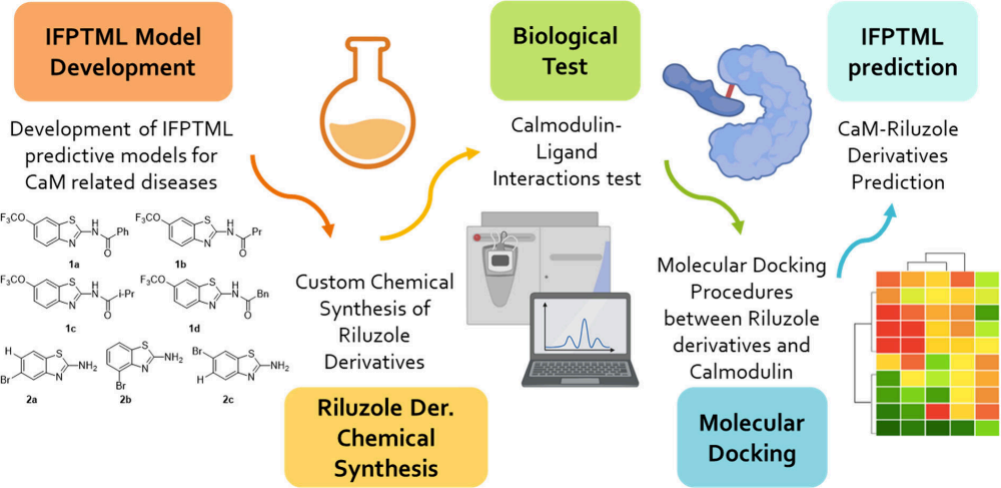
General workflow of the project.

### 4.1 IFPTML Model Development

IFPTML analysis involved four phases: the IF process, PT variability quantification, and AI/ML algorithm training, validation, and use. In the initial IF phase, data gathering, data curation and data preprocessing tasks were carried out. Importantly, system conceptualization is conceptual decomposition of the system in different subsystems that are easy to study. In this case, the system was theoretically divided into two subsystems: drug information related to assays and protein data related to assays. Taking this into account, databases were examined and processed. Continuing the IFPTML process, in the PT phase, the reference function and perturbation theory operators (PTO) or moving averages (MA) were calculated, which are used to quantify all the perturbations/variability on the input variables for all subsystems of the query system with respect to conditions or labels for the systems of reference. Lastly, the ML-Phase involved the training and validation of different ML models.^6–9,11,67^ The general procedure followed in this part can be seen schematically in the Figure 12.

**Figure 12:**
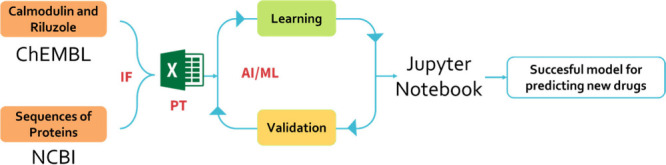
Workflow of the IFPTML model development.

#### 4.1.1 IF Phase: Construction and Processing of the Database

First, the ChEMBL web page was used to build the data set. The search was carried out searching for the different proteins related to CaM inside the Ca^2+^ signaling pathway. To do so, the Ca^2+^ signaling pathway was searched on the KEGG encyclopedia,^14^ and all the proteins directly related to CaM were listed (Figure 1). ChEMBL was then used to download all the preclinical assays where the target proteins were these ones. Responses of CaM- and riluzole-related compounds were obtained, having approximately 4000 trials (Supporting Information II, Table S1). Each *v_ij_* value depends on the analyzed compound and the conditions used to carry out the test (*c_j_* = *c*_0_, *c*_1_, *c*_2_, ..., *c_n_*).

Furthermore, once the chemical compound data was obtained from ChEMBL, the NCBI web page was used to download the data about the proteins analyzed in each test. GenBank together with Protein BLAST^27,28^ tools were used to get the FASTA sequence and three biological active domains, respectively (Supporting Information II, Table S2). Thus, the protein operators were added to the developed chemoinformatic model.

The descriptors used to calculate the operators of the cheminformatics model can be classified into two groups: drug descriptors (*D*_k_(drug)) (Supporting Information II, Table S3) and protein descriptors (*D*_*k*_(prot, dom)) (Supporting Information II, Table S4). On the one hand, drug descriptors (*D*_*k*_(drug)) differed in chemical compound molecular weight (MW), Lipinski’s rule of five (LRO5), interatomic electronegativity, van der Waals surface area (PSA) and *n*-octanol/water partition coefficient (Log *P*). The concentration of the inhibitor used as a control and the concentration of the substrate utilized in the tests were also labeled as variables (V_ki_). And, on the other hand, for the biologically active domains in the protein descriptors (*D*_*k*_(prot,dom)), MARCH-INSIDE 2.0 (*Markovian Chemicals in Silico Design*) program^41,29,42,30^ was used in order to get “proteins-nandy-polarity-acidity-plot” representations. These representations encode the physicochemical properties of amino acids—such as electronegativity, polarity, and acidity—along the protein sequence. MARCH-INSIDE then applies a Markovian formalism to propagate these properties across the residue network in each domain, producing quantitative descriptors that capture domain-level physicochemical signatures. These numerical values are then used as inputs in the IFPTML model.

#### 4.1.2 PT Phase: Estimation of Perturbation Theory Operators and Deltas

Once the descriptors of the data series were collected, the Perturbation Theory Operators^68^ (PTOs) were calculated. In this work, to quantify the perturbation, the method used was based on Box-Jenkins^43^ Moving Average (MA) operators. The expected value for the reference system is usually measured as the average value of the molecular descriptor for all cases in the database measured under the same conditions, *c*_*j*_ (⟨*D*_*k*_(*c*_*j*_)⟩). Besides, as crucial point, this model incorporates assays that were tested under multiple boundary conditions at the same time as commented on Table 2. The MA operators were divided into two groups, based on the following boundary conditions: the first group of MA operators took into account the assay conditions *c*_assay_ = (*c*_1_, *c*_2_, *c*_3_, *c*_4_, *c*_5_); whereas the second group of MA operators were calculated using the remaining data conditions, *c*_dat_ = (*c*_6_, *c*_7_, *c*_8_, *c*_9_, *c*_10_). Consequently, the moving average values (⟨*D*_k_(*c*_*j*_)⟩) were calculated for those assays under the same boundary conditions, *c*_assay_ and *c*_dat_ (Supporting Information II, Table S5 and S6). Furthermore, the delta values are based on how far the actual value of the assay is from the average value, under the same boundary conditions. Accordingly, the calculation of the delta values (Δ*Dk*(*c*_*j*_)) of drugs and proteins were completed using the following eq (Supporting Information II, Table S7):

2 $$\Delta D_{k}\left(c_{j}\right)=D_{k}-\langle D_{k}\left(c_{j})\right\rangle$$

#### 4.1.3 Calculation of the Output Variable and Reference Function

There are multiple classes of *v_ij_* (output parameters) for drug activity measured in the assay, e.g., IC_50_, *K_i_*, *K_m_*, etc. The values *v_ij_* compiled were not values of the same kind in many cases. That is why classification techniques were used. To know whether the shown value is the expected value for that activity measurement, the desirability parameter was defined for each case. The desirability displays whether the output parameter is expected to be maximized or minimized, considering its biological nature. Thus, two different cases were to be expected: when the output parameter was expected to maximize its value, the desirability would be *d*(*c*_0_) = +1; on the contrary, when the output parameter was expected to minimize its value, then, the desirability would be *d*(*c*_0_) = −1. For instance, the properties with units of percentage (residual activity, inhibition, metabolism of the drug, and activity) have been adjusted to *d*(*c*_0_) = +1 value, since they are mostly inhibition or activity of the drug, because it is advisible to maximize the percentage. And, on the other hand, the activity with units of concentration (IC_50_, *K_i_*, potency, *K_d_*, and *K_m_*) were assigned a value of *d*(*c*_0_) = −1, to achieve the desired effect by decreasing the concentration of the drug, as the compound will be more active.

The values of *v_ij_* were then transformed into Boolean variables *f*(*v_ij_*)_obj_ = 1 or 0. These values are vital to be able to distinct between the active compounds (*f*(*v_ij_*)_obj_ = 1) and the inactive ones (*f*(*v_ij_*)_obj_ = 0). The values of *f*(*v_ij_*)_obj_ came from different cut-offs that were decided for each drug activity (*c*_0_). For activity measures expressed in concentration units (e.g., IC_50_, *K_i_*), a cutoff of 100 nM was used, reflecting standard potency criteria. In the case of inhibition percentages, a 70% threshold was applied, following common screening practices. For other assay types lacking standard thresholds, the data set mean was used as a reference point. According to those cut-offs and considering the desirability commented before, if the data was above the cutoff and the desirability was *d*(*c*_0_) = +1, then, *f*(*v_ij_*)_obj_ = 1, and *f*(*v_ij_*)_obj_ = 0 otherwise. On the other hand, if the data was below the cutoff and the desirability was set as *d*(*c*_0_) = −1, then, *f*(*v_ij_*)_obj_ = 1, and *f*(*v_ij_*)_obj_ = 0 otherwise. Table S6 in Supporting Information I sums up the desirability and cutoff for each case.

To finish with the treatment of the data series, the calculation of the input variable was carried out: the calculation of the reference function (*f*(*v_ij_*)_ref_). This value shows the probability of a compound to be active under certain boundary conditions before making predictions. Therefore, it is calculated dividing the number of active compounds (*f*(*v_ij_*)_obj_ = 1) with the number of total active and inactive compounds that meet the same conditions (Supporting Information II, Table S8):

3 $$f(v_{ij}{)}_{\mathrm{ref}}=p({f\left(v_{ij}/c_{j}\right)}_{\mathrm{obj}}=1{)}_{\mathrm{ref}}=\frac{n\left({f\left(v_{ij}/c_{j}\right)}_{\mathrm{obs}}=1\right)}{n\left({f\left(v_{ij}/c_{j}\right)}_{\mathrm{obs}}\right)}$$

#### 4.1.4 ML Phase: Creation of the IFPTML Model

To develop a robust chemoinformatic predictive model, both linear and nonlinear classification approaches were applied to the processed data set (see Supporting Information II, Table S9). First, the Linear Discriminant Analysis (LDA) model, a linear classifier (IFPTML-LDA), was implemented following the framework of eq 4. This model allowed for the calculation of scoring function values *f*(*v_ij_*)_calc_ for each *i*th compound across a variety of preclinical assay (*j*) conditions *c*_*j*_ = (*c*_0_, *c*_1_, *c*_2_, ..., *c*_*j*max_). Likewise, the model’s boundary conditions are presented as *c*_*j*_ vectorial parameters by way of *c*_*j*_ = *c*_assay_ U *c*_dat_ = (*c*_1_, *c*_2_, *c*_3_, *c*_4_, *c*_5_) U (*c*_6_, *c*_7_, *c*_8_, *c*_9_, *c*_10_) = (*c*_1_, *c*_2_, *c*_3_, ..., *c*_10_), where *c*_assay_ = (*c*_1_, *c*_2_, *c*_3_, *c*_4_, *c*_5_), *c*_dat_ = (*c*_6_, *c*_7_, *c*_8_, *c*_9_, *c*_10_).

4 $$f(v_{ij}{)}_{\mathrm{calc}}=a_{0}+a_{1}\cdot {f\left({v_{\mathrm{drug}}}_{i},c_{j}\right)}_{\mathrm{ref}}+\overset{k_{\max}}{\underset{k=1}{\sum }}b_{k}\cdot D_{k}\left({\mathrm{drug}}_{i}\right)+\overset{k_{\max}}{\underset{k=1}{\sum }}d_{k}\cdot D_{k}\left({\mathrm{prot}}_{t},{\mathrm{dom}}_{m}\right)+\overset{k_{\max},j_{\max}}{\underset{k=1,j=0}{\sum }}{b^{\prime }}_{k}\cdot \Delta D_{k}\left(\mathrm{drug},{c^{\prime }}_{j}\right)+\overset{k_{\max},j_{\max}}{\underset{k=1,j=0}{\sum }}{d^{\prime }}_{k}\cdot \Delta D_{k}\left({\mathrm{prot}}_{t},{\mathrm{dom}}_{m},{c^{\prime }}_{j}\right)$$

For the model to be deemed acceptable, accuracy and sensitivity parameters must be above 70%, with a balance between data classifications. Apart from the LDA model range of nonlinear models were developed and implemented. The nonlinear models tested included Random Forest, Support Vector Machine (SVM with RBF kernel), Decision Tree, K-Nearest Neighbors (KNN), Gradient Boosting, and XGBoost.

Python programming language was used to develop all the models, using the scikit-learn and pandas for data manipulation.^44^ The data set was split into 80% training and 20% test using the train_test_split function, and models were evaluated for an acceptable threshold of over 70% accuracy and sensitivity with balanced classifications. Each model offered unique ways to capture complex relationships within the data. To enhance model accuracy, hyperparameters were optimized through Grid Search and cross-validation. Model performance was assessed through various metrics, including accuracy, sensitivity, specificity, AUC-ROC, and confusion matrices. The workflow was executed in a Jupyter Notebook to facilitate reproducibility and efficient experimentation.

### 4.2 Case Study of Riluzole Derivatives

As part of this work, additional tasks were undertaken, including a focused case study on riluzole derivatives. This study involved several key steps: first, the synthesis of riluzole derivatives; second, the evaluation of their activity in a biological assay designed to monitor Ca^2+^-dependent interactions between CaM and target proteins. To further understand these interactions, a docking study was conducted for comparison. Finally, the previously developed predictive model was applied to assess the efficacy of the riluzole derivatives and to demonstrate its practical application.

#### 4.2.1 Synthesis of Riluzole Derivatives

The synthesis of various riluzole derivatives was undertaken as the next step. Riluzole was selected as the key compound due to its established role in modulating Ca^2+^ signaling and its potential therapeutic effects on CaM-related diseases. Based on this, seven different riluzole derivatives were synthesized for further study. N-acyl riluzole derivatives (**1a**–**c**) were prepared via Schotten–Baumann reaction using anhydrides and DIPEA in DMF, followed by purification through column chromatograph.^69^ The N-benzoyl derivative (**1d**) was synthesized via the Steglich reaction using benzoic acid, DCC, and a catalytic amount of DMAP.

Additionally, brominated benzo­[d]­thiazol-2-amines (**2a**–**e**) were synthesized following the Stuckwisch procedure^70^ by reacting bromoanilines with KSCN and bromine in acetic acid. The initially formed thioureas underwent cyclization to yield brominated benzothiazoles in moderate yields.^71^ See Supporting Information I, Section 6 for further explanation.

#### 4.2.2 Biological Assay of Riluzole Derivatives

To investigate the interactions between CaM and target proteins, advanced techniques were employed to ensure precise monitoring of these interactions. One such technique involves biosensors based on Förster FRET, designed to track Ca^2+^-dependent interactions between CaM and its ligands.^22^ These biosensors consist of CaM fused to the target protein and two fluorophores a donor and acceptor at the N- and C-termini, enabling real-time tracking.^22,63^ Fusion proteins were purified using size-exclusion chromatography. Additionally, the biosensors were produced through expression and purification of fusion proteins, followed by size-exclusion chromatography to isolate monomeric fractions. FRET measurements, performed using a fluorimeter, quantified Ca^2+^-dependent changes in fluorescence emissions, with drug titrations used to test interaction dynamics. Refer to Supporting Information I, Section 7 for additional details.

#### 4.2.3 Docking Study

To further investigate the molecular interactions between riluzole derivatives and their target proteins, docking simulations were employed. This computational approach allows for the prediction of binding affinities and the identification of potential interaction sites, providing deeper insights into the efficacy and specificity of the synthesized compounds. For this analysis, the ligands and the receptor were prepared with LigPrep^72^ and the Protein Preparation Wizard,^72^ respectively. The crystal structure of the SK4 channel from^65^ was used as the receptor for the docking simulations. Specifically, a 15 Å × 15 Å × 15 Å grid-centered known riluzole binding pocket formed between the CaM N-lobe and the S45A helix of the SK4 channel was used as the search space for the docking simulation. Docking was performed using Glide^73^ in SP mode. Additional information can be found in Supporting Information I, Section 8.

#### 4.2.4 IFPTML Predictive Study

One of the main uses of the predictive model is to predict whether a molecule could score a better value of *v_ij_* biological activity on the *j* ^th^ preclinical assay, as long as there is a reference molecule to compare it with. In this context, it was decided to use the IFPTML-XGB model to predict the output of different proposed compounds (riluzole derivatives), having riluzole as reference, against CaM and its related kinases. The aim of this part was to verify if the riluzole derivatives bonded to CaM better or similarly than riluzole. To do so, the derivatives and assay’s information was integrated, the perturbation theory operators were calculated and predictions were generated using the IFPTML-XGB classification model (Figure 13). For more details, consult Supporting Information I, Section 9.

**Figure 13:**
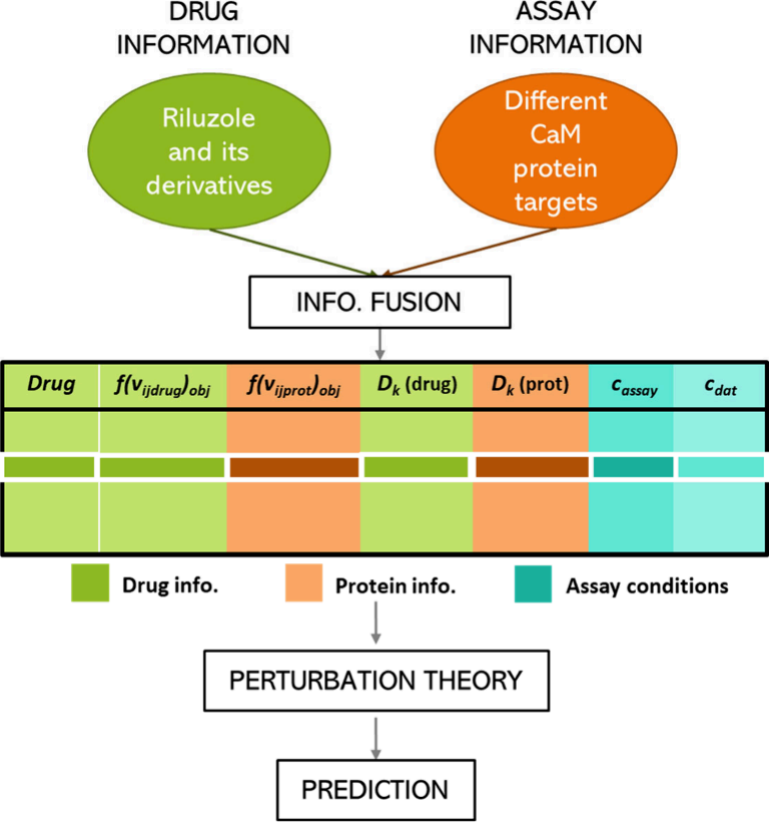
Workflow of IFPTML prediction of new riluzole derivatives against CaM.

## Supplementary Material





## Data Availability

All data and code used in this study are publicly available at the GitHub repository: https://github.com/maiderbaltasar/CaMIFPTML. The repository includes the training and validation data set (Readable Data_Python.csv) in machine-readable CSV format, as required by JCIM guidelines.^45^ It also contains the Jupyter notebook for model development (camptml.ipynb), figures, and extended supporting materials (Supporting Information II). The repository has been archived in Zenodo for long-term access and citation: 10.5281/zenodo.15423309.

## Notes

Supporting tables (ZIP)
Additional information on biological studies, copies of ^1^H and ^13^C NMR spectra for new compounds reported (**1a**–**2d**) (PDF)
