# Supplementary material for: Machine Learning Approach for Predicting Drug-Like Molecules Targeting Calmodulin Pathway Proteins
Source: J Chem Inf Model. 2025 Oct 22;65(21):11892–907. doi: 10.1021/acs.jcim.5c02111 (PMC12606648; doi:10.1021/acs.jcim.5c02111)
Supplement: Supplementary file 2 [file ci5c02111_si_002.pdf]

Supporting Information I. A machine learning approach for predicting drug-like molecules targeting Calmodulin pathway proteins

*Maider Baltasar-Marchueta,[1,2] Naia López,[1] Sara Alicante,[3] Iratxe Barbolla,[1] Markel Garcia Ibarluzea,[4,5] Rafael Ramis,[4,5] Ane Miren Salomon,[1] Arantza Muguruza-Montero,[3] Eider Nuñez Viadero,[3] Aritz Leonardo,[4,5] Sonia Arrasate,[1] Nuria Sotomayor,[1] Matthew M Montemore,[2] Alvaro Villarroel,[*3] Aitor Bergara,[*4,5] Esther Lete,[*1] and Humberto González-Díaz[*1,3,6]*

[1] Department of Organic and Inorganic Chemistry, University of the Basque Country UPV/EHU, Barrio Sarriena, s/n, 48940 Leioa, Bizkaia, Spain.

[2] Department of Chemical and Biomolecular Engineering, Tulane University, 6823 St Charles Avenue, New Orleans, Louisiana 70118, United States.

[3] Biofisika Institute, CSIC-UPV/EHU, Barrio Sarriena, s/n, 48940 Leioa, Bizkaia, Spain.

[4] Donostia International Physics Center, Manuel Lardizabal Ibilbidea, 4, 20018 Donostia, Gipuzkoa, Spain.

[5] Department of Physics and EHU Quantum Center, University of the Basque Country UPV/EHU, Barrio Sarriena, s/n, 48940 Leioa, Bizkaia, Spain.

[6] IKERBASQUE, Basque Foundation for Science, Euskadi Pl., 5, Abando, 48009 Bilbao, Bizkaia, Spain.

1. IFPTML models additional results

1.1. IFPTML LDA model

Table S1. Information about input variables in the best model found.

Operator Equation	Operator Information
$\Delta D_{024}(drug_i,c_{dat}) = D_{024}(drug_i) - < D_{024}(drug_i,c_{dat}) >$	PTO[a] for electronegativity attribution of drug's heteroatoms at level 0
$\Delta D_{099}(drug_i,c_{dat}) = D_{099}(drug_i) - < D_{099}(drug_i,c_{dat}) >$	PTO[a] for AlogP attribution of drug's heteroatoms at level 5
$\Delta D_{099}(drug_i,c_{assay}) = D_{099}(drug_i) - < D_{099}(drug_i,c_{assay}) >$	PTO[a] for AlogP attribution of drug's heteroatoms at level 5
$\Delta D_{024}(drug_i,c_{assay}) = D_{024}(drug_i) - < D_{024}(drug_i,c_{assay}) >$	PTO[a] for electronegativity attribution of drug's heteroatoms at level 0
$f(v_{ij})_{ref} = n(f(v_{ij} = 1,c_0)/n_{c_0,a}$	A priori probability that a compound that interacts with a protein expressed in one of the brain regions of interest is active, for the specific condition c_0.
$\Delta D_{089}(drug_i,c_{dat}) = D_{089}(drug_i) - < D_{089}(drug_i,c_{dat}) >$	PTO[a] for AlogP attribution of drug's halogen atoms at level 1
$\Delta D_{089}(drug_i,c_{assay}) = D_{089}(drug_i) - < D_{089}(drug_i,c_{assay}) >$	PTO[a] for AlogP attribution of drug's halogen atoms at level 1
$\Delta D_{030}(drug_i,c_{dat}) = D_{030}(drug_i) - < D_{030}(drug_i,c_{dat}) >$	PTO[a] for electronegativity attribution of drug's labile hydrogen atoms at level 0
$\Delta D_{030}(drug_i,c_{assay}) = D_{030}(drug_i) - < D_{030}(drug_i,c_{assay}) >$	PTO[a] for electronegativity attribution of drug's labile hydrogen atoms at level 0
$\Delta D_{093}(drug_i,c_{dat}) = D_{093}(drug_i) - < D_{093}(drug_i,c_{dat}) >$	PTO[a] for AlogP attribution of drug's halogen atoms at level 5

$\Delta D_{093}(drug_i, c_{assay}) = D_{093}(drug_i) - < D_{093}(drug_i, c_{assay}) >$	PTO[a] for AlogP attribution of drug's halogen atoms at level 5
$\Delta D_{095}(drug_i, c_{dat}) = D_{095}(drug_i) - < D_{095}(drug_i, c_{dat}) >$	PTO[a] for AlogP attribution of drug's heteroatoms at level 1
$\Delta D_{095}(drug_i, c_{assay}) = D_{095}(drug_i) - < D_{095}(drug_i, c_{assay}) >$	PTO[a] for AlogP attribution of drug's heteroatoms at level 1
$\Delta D_{100}(drug_i, c_{dat}) = D_{100}(drug_i) - < D_{100}(drug_i, c_{dat}) >$	PTO[a] for AlogP attribution of drug's labile hydrogen atoms at level 0
$\Delta D_{100}(drug_i, c_{assay}) = D_{100}(drug_i) - < D_{100}(drug_i, c_{assay}) >$	PTO[a] for AlogP attribution of drug's labile hydrogen atoms at level 0
$\Delta D_{031}(drug_i, c_{dat}) = D_{031}(drug_i) - < D_{031}(drug_i, c_{dat}) >$	PTO[a] for electronegativity attribution of drug's labile hydrogen atoms at level 1
$\Delta D_{083}(drug_i, c_{assay}) = D_{083}(drug_i) - < D_{083}(drug_i, c_{assay}) >$	PTO[a] for AlogP attribution of drug's unsaturated carbons atoms at level 1
$\Delta D_{031}(drug_i, c_{assay}) = D_{031}(drug_i) - < D_{031}(drug_i, c_{assay}) >$	PTO[a] for electronegativity attribution of drug's labile hydrogen atoms at level 1
$\Delta D_{007}(drug_i, c_{assay}) = D_{007}(drug_i) - < D_{007}(drug_i, c_{assay}) >$	PTO[a] for electronegativity attribution of drug's saturated carbon atoms at level 1
$\Delta D_{087}(drug_i, c_{assay}) = D_{087}(drug_i) - < D_{087}(drug_i, c_{assay}) >$	PTO[a] for AlogP attribution of drug's unsaturated carbon atoms at level 5

[a)] Each PTO calculates how much the value of the descriptor deviates from the average value over the dataset, for the conditions c_{assay} or c_{dat}.

1.2. IFPTML non-linear models

To evaluate and compare the performance of different machine learning models, we implemented K-Fold Cross-Validation[1] with 10 folds. This technique involves splitting the dataset into ten subsets, training the model on 9 of them, and validating it on the remaining one, repeating this process 10 times so that each fold serves as a test set once. The average accuracy and receiver operating characteristic-area under the curve (ROC AUC) across all folds provide

a more reliable estimate of model performance. We tested six different non-linear models, and their mean accuracy and mean ROC AUC from cross-validation were recorded:

Table S2. Mean accuracy and mean ROC AUC of each of the non-linear models.

	Mean Accuracy (%)	Mean ROC AUC
RF	87.22	0.94
SVM	76.15	0.85
DT	84.47	0.84
KNN	80.42	0.88
GB	84.92	0.93
XGB	88.50	0.95

In the variable selection process, the first step involved calculating the correlation matrix to evaluate the relationships between features. A heatmap was generated (Figure S2) to visually represent the matrix, highlighting pairs of features with strong correlations ($|r|>0.97$). Features exhibiting high correlations were identified as redundant, and one feature from each highly correlated pair was removed to minimize multicollinearity and redundancy in the dataset. The second step focused on variance thresholding to remove features with low variability. A variance threshold of 0.01 was applied, maintaining only features with sufficient variability. This process reduced the number of features from 252 to 71, achieving a significant reduction in dimensionality.

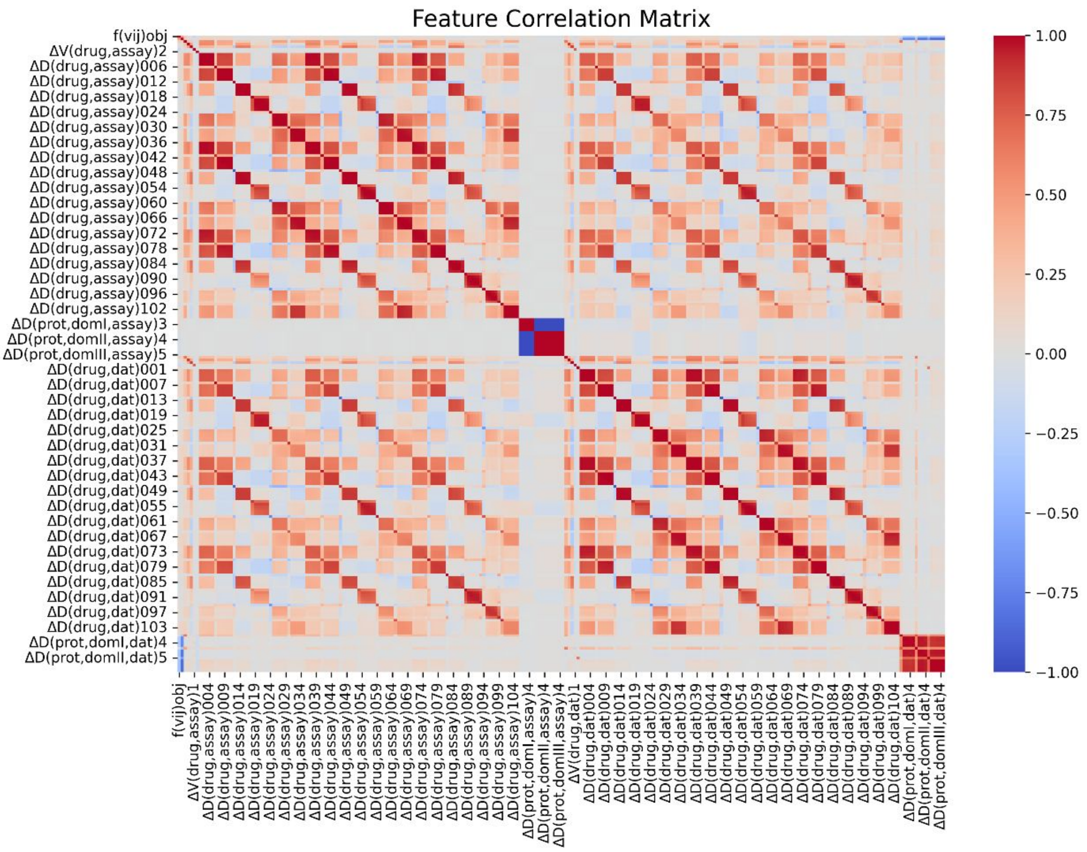

Figure S1. Feature correlation matrix heatmap.

Table S3. Hyperparameters of IFPTML-XGB used in the model after hyperparameter tunning.

Hyperparameter	Initial Model (Default)	After RandomizedSearchCV	After GridSearchCV
n_estimators	100	170	160
max_depth	6	10	12
learning_rate	0.3	0.2678	0.2678
subsample	1.0	1.0	1.0
colsample_bytree	1.0	0.6	0.6
gamma	0	0.1	0.1
min_child_weight	1	5	5

Table S4. Information about top 20 input variables in the XGB ML model.

Operator Equation	Operator Information
$f(v_{ij})_{ref} = n(f(v_{ij} = 1, c_0)/n_{c_0,a}$	A priori probability that a compound that interacts with a protein expressed in one of the brain regions of interest is active, for the specific condition c_0.
$\Delta V_2(drug_i, c_{assay}) = V_2(drug_i) - < V_2(drug_i, c_{assay}) >$	PTO[a] for inhibitor concentration
$\Delta D_2(drug_i, c_{dat}) = D_2(drug_i) - < D_2(drug_i, c_{dat}) >$	PTO[a] for Lipsinki's rule of five
$\Delta D_{053}(drug_i, c_{dat}) = D_{053}(drug_i) - < D_{053}(drug_i, c_{dat}) >$	PTO[a] for van der Waals forces of drug's halogen atoms at level 0
$\Delta D_2(prot_t, dom_I, c_{dat}) = \Delta D_2(prot_t, dom_I) - < \Delta D_2(prot_t, dom_I, c_{dat}) >$	PTO[a] for electronegativity of the protein at the first domain at level 2
$\Delta V_1(drug_i, c_{dat}) = V_1(drug_i) - < V_1(drug_i, c_{dat}) >$	PTO[a] for substrate concentration
$\Delta D_{018}(drug_i, c_{assay}) = D_{018}(drug_i) - < D_{018}(drug_i, c_{assay}) >$	PTO[a] for electronegativity attribution of drug's halogen atoms at level 0
$\Delta D_1(drug_i, c_{dat}) = D_1(drug_i) - < D_1(drug_i, c_{dat}) >$	PTO[a] for molecular weight
$\Delta D_{059}(drug_i, c_{dat}) = D_{059}(drug_i) - < D_{059}(drug_i, c_{dat}) >$	PTO[a] for Van der Waals forces of drug's heteroatoms at level 0
$\Delta D_1(prot_t, dom_{II}, c_{dat}) = D_1(prot_t, dom_{II}) - < D_1(prot_t, dom_{II}, c_{dat}) >$	PTO[a] for electronegativity of the protein at second domain at level 1
$\Delta D_{083}(drug_i, c_{dat}) = D_{083}(drug_i) - < D_{083}(drug_i, c_{dat}) >$	PTO[a] for AlogP attribution of drug's unsaturated carbon atoms at level 1
$\Delta D_{100}(drug_i, c_{dat}) = D_{100}(drug_i) - < D_{100}(drug_i, c_{dat}) >$	PTO[a] for AlogP attribution of drug's hydrogen atoms at level 0
$\Delta D_{101}(drug_i, c_{assay}) = D_{101}(drug_i) - < D_{101}(drug_i, c_{assay}) >$	PTO[a] for AlogP attribution of drug's hydrogen atoms at level 5
$\Delta D_{031}(drug_i, c_{dat}) = D_{031}(drug_i) - < D_{031}(drug_i, c_{dat}) >$	PTO[a] for electronegativity attribution of drug's hydrogen atoms at level 1
$\Delta D_{018}(drug_i, c_{dat}) = D_{018}(drug_i) - < D_{018}(drug_i, c_{dat}) >$	PTO[a] for electronegativity attribution of drug's halogen atoms on level 0

$\Delta V_1(drug_i, c_{assay}) = V_1(drug_i) - \langle V_1(drug_i, c_{assay}) \rangle$	PTO[a] for substrate concentration
$\Delta D_2(drug_i, c_{assay}) = D_2(drug_i) - \langle D_2(drug_i, c_{assay}) \rangle$	PTO[a] for Lipsinki's rule of five
$\Delta D_{101}(drug_i, c_{dat}) = D_{101}(drug_i) - \langle D_{101}(drug_i, c_{dat}) \rangle$	PTO[a] for AlogP attribution of drug's hydrogen atoms at level 1
$\Delta D_{054}(drug_i, c_{dat}) = D_{054}(drug_i) - \langle D_{054}(drug_i, c_{dat}) \rangle$	PTO[a] for van der Waals forces of drug's halogen atoms at level 1
$\Delta D_{087}(drug_i, c_{dat}) = D_{087}(drug_i) - \langle D_{087}(drug_i, c_{dat}) \rangle$	PTO[a] for AlogP attribution of drug's unsaturated carbons atoms at level 5

[a)] Each PTO calculates how much the value of the descriptor deviates from the average value over the dataset, for the conditions c_{assay} or c_{dat}.

2. Synthesis of riluzole derivatives additional results

The next schemes sumirez the experimental reactions used to sythesize riluzole derivatives.

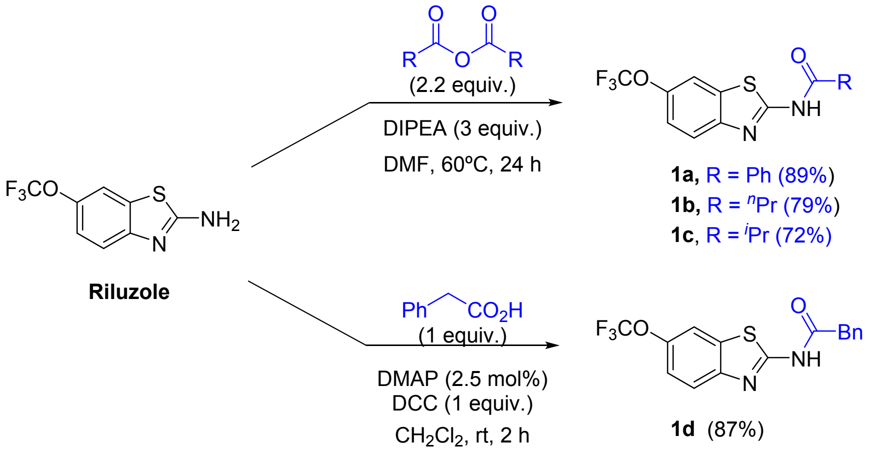

Scheme S1. Synthesis of N-acylated riluzole derivatives **1a**-d.

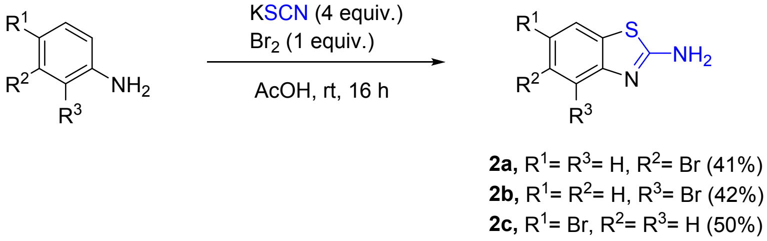

Scheme S2. Synthesis of brominated riluzole derivatives **2a**-c.

3. Biological assay of riluzole derivatives additional results

Table S5. Relative amplitude in the presence of the indicated drugs at 100 µM with the YC-Nano15 biosensor. Numbers represent mean ± standard error of the mean (SEM) (n ≥ 3). Total inhibition is the relative reduction of Phase I and Phase II amplitudes combined.

Compound	Total inhibition
Riluzole	0.3 ± 1.3
**1a**	8.0 ± 2.2
**1b**	2.0 ± 1.4
**1c**	-3.7 ± 1.9
**1d**	-0.1 ± 0.4
**2a**	-6.7 ± 0.8
**2b**	-0.1 ± 0.8
**2c**	-3.5 ± 1.4

4. Molecular Docking study additional results

The docked conformations of ligands **1a** and **1b** are similar to riluzole's, as their $-OF_3$ group is also pointing towards the hydrophobic pocket. Similarly, the substituents added to these derivatives are located at the opening of the binding pocket, and, in both cases, the amine groups maintain their hydrogen bonds with E54. Ligands **1c** and **1d** have opposite orientations to riluzole and the **1a** and **1b** derivatives, with their $-OF_3$ group pointing towards the entrance of the binding pocket, and the groups substituting the amino group, which for both of these ligands are hydrophobic, buried in the hydrophobic pocket between CaM and SK4. Meanwhile, ligands **2a**, **2b** and **2c**, which do not contain the hydrophobic $-OF_3$ group from riluzole, and are also

smaller in volume, are positioned deeper in the hydrophobic pocket compared to the other molecules, and do not interact with the entrance of the binding pocket.

We validated the conformations obtained through docking by performing binding pose metadynamics (BPMD) simulations [2]. During the last 2 ns of the simulation, the portion of preserved native non-covalent contacts between ligand and protein and the root-mean-square deviation (RMSD) of the ligand heavy atoms after alignment of the protein Cα atoms were collected. Based on the collected RMSD data, the PoseScore is computed, whereas the preserved contact data is used to compute the ContactScore. The stability of the predicted binding poses was then assessed by computing the CompScore, which combines both the PoseScore and the ContactScore. Figure S3 shows the CompScores for each ligand studied in this work, as well as the PoseScores and ContactScores from which they are calculated. For the docked conformations analyzed through BPMD, we observe that all of them display behaviors of stable conformations, with an average RMSD below 2 Å.

To further assess the stability of these poses over longer timescales, we also conducted 100 ns conventional molecular dynamics (MD) simulations for each ligand-protein complex. The systems were prepared using CHARMM-GUI, embedding the full SK2 structure within a POPC lipid bilayer, solvated with TIP3P water, and neutralized with 150 mM KCl. The CHARMM36 force field was used for the protein, lipid and ligands. All MD simulations were performed using GROMACS 2024.4.

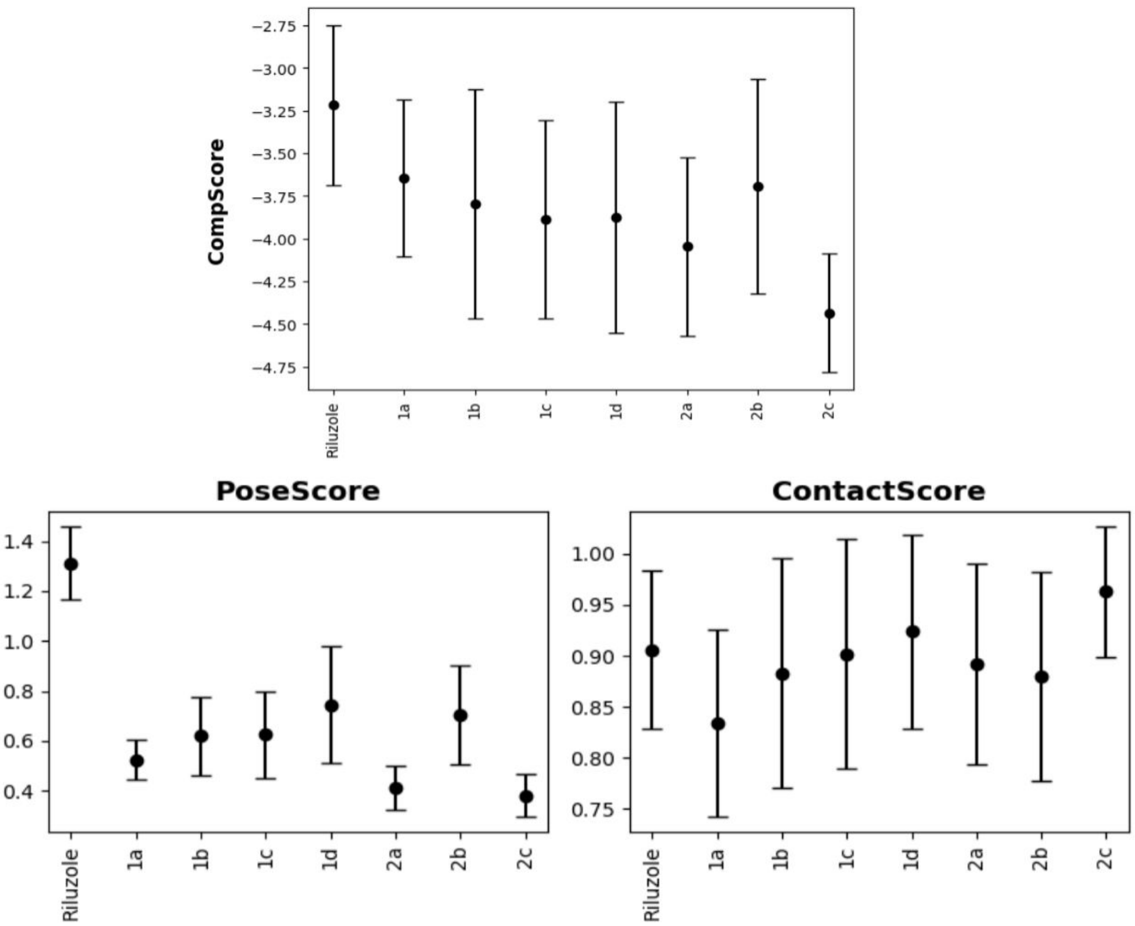

Figure S2. Mean and standard deviation of the PoseScore, ContactScore, and CompScore measured for riluzole and all its derivatives. The scores were calculated from the last 2 ns of the metadynamics simulations, averaged over 10 short simulations.

To further assess the stability of these poses over longer timescales, we also conducted 100 ns conventional molecular dynamics (MD) simulations for each ligand-protein complex. The systems were prepared using CHARMM-GUI, embedding the full SK2 structure within a POPC lipid bilayer, solvated with TIP3P water, and neutralized with 150 mM KCl. The CHARMM36 force field was used for the protein, lipid and ligands. All MD simulations were performed using GROMACS 2024.4.

Analysis of these extended simulations shows patterns consistent with the initial BPMD results (figure S4A and S4B). The ligand root-mean-square deviation (RMSD) and the preservation of key ligand-protein contacts throughout the 100 ns trajectories suggest that all the predicted poses are stable.

Furthermore, the MD trajectories were used to estimate relative binding free energies using the MM-PBSA method as implemented in the g_mmpbsa software.[3] Snapshots sampled every 1ns from the equilibrated portion of each trajectory were used to compute the free energy estimates. The results show a reasonable agreement with the experimental inhibition data, particularly in terms of rank correlation (figure S4C). This suggests that the predicted poses are not only stable but can also serve as a structural basis for quantitative estimations of ligand potency.

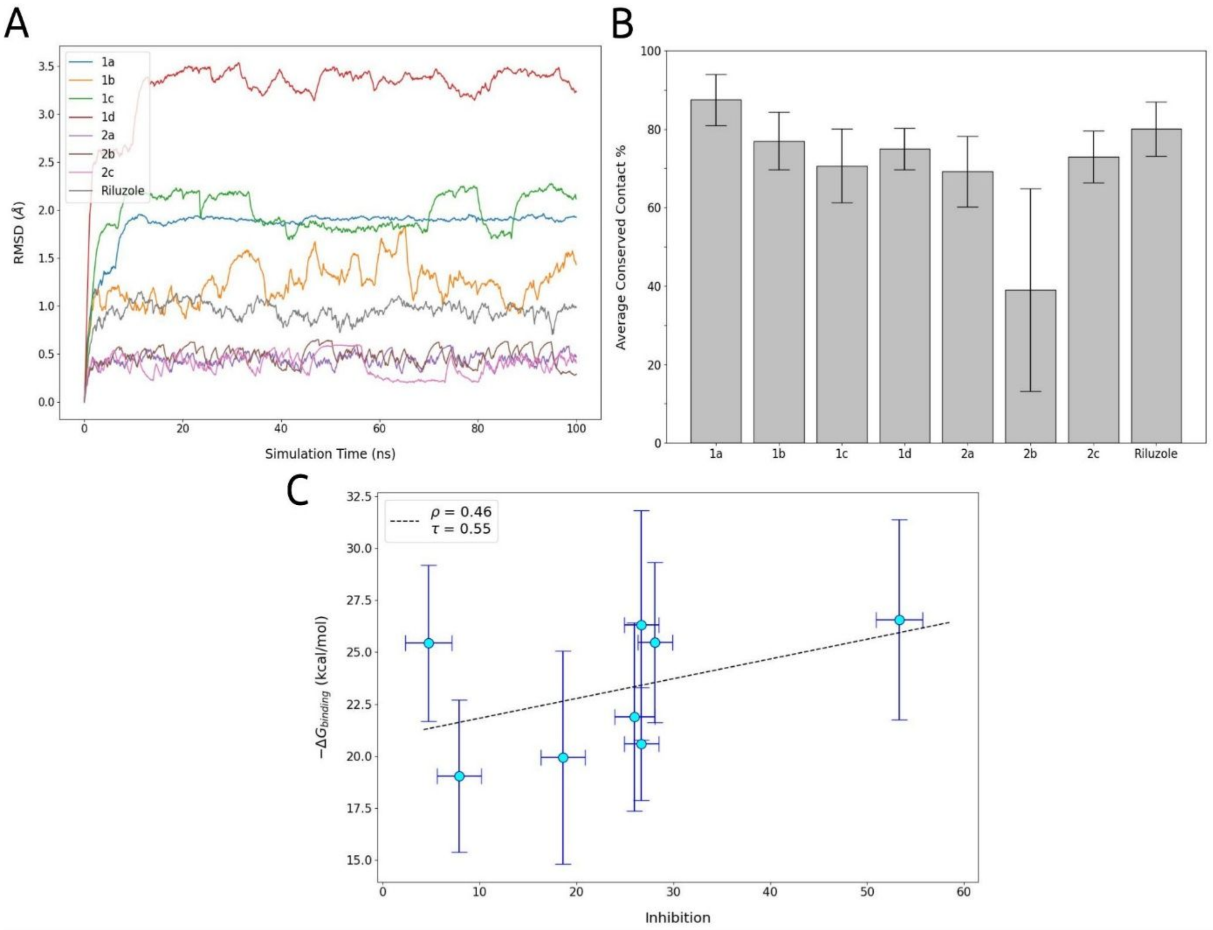

Figure S3. Analysis of 100ns molecular dynamics simulations started from docking poses. (A) RMSD of each ligand with respect to the conformation predicted through docking. (B) Percentage of native contacts, defined as residues within a 4.5Å distance threshold of the ligand, that are preserved throughout the simulation, averaged over all collected frames. (C) Correlation, measured in terms of Pearson correlation (ρ) and Kendall rank correlation (τ), between the experimental inhibition and binding free energies calculated using MM-PBSA for all ligands.

5. IFPTML model development additional details

Table S6. Cut-off and desirability for each drug activity.

**Drug activity measured (c_0)**	**Cut-off**	**d(c_0)**
Residual activity (%)	70,00	1
IC_{50} (nM)	100,00	-1
K_i (nM)	100,00	-1
Inhibition (%)	70,00	1
Potency (nM)	100,00	-1
K_i Ratio	432,00	1
Drug metabolism (%)	70,00	1
$T_{1/2}$ (h)	4,00	-1
K_d (nM)	100,00	-1
Activity (%)	70,00	1
Kinetics (min^{-1})	0,17	1
K_m (nM)	100,00	-1
Relative potency	3,60	1

6. Synthesis of riluzole derivatives additional details

All commercial chemicals were reagent grade and were used without further purification unless otherwise specified. All solvents used in reactions were anhydrous and purified according to standard procedures. All air- or moisture-sensitive reactions were performed under argon; the glassware was dried (130 °C) and purged with argon. TLC was carried out with 0.2 mm-thick silica gel Merck F254 plates. Visualization was accomplished by UV light (λ = 254 nm and 360 nm). Flash column chromatographic separations and purifications were performed on silica Flash P60 (Silicycle), 230-400 mesh ASTM. Final compounds were purified to ≥95% purity as assessed by ^{1}H NMR spectra and analytical liquid chromatography. Melting points

were measured in a Büchi B-540 apparatus in unsealed capillary tubes. IR spectra were obtained using Attenuated Total Reflection (ATR) in a JASCO FT/IR 4100 in the interval between 4000 and 400 cm^{-1} with a 4 cm^{-1} resolution. Only characteristic bands are given in each case. ^{1}H and ^{13}C NMR spectra were recorded at 20-25 ˚C on either a Bruker AC-300 spectrometer (300 MHz for ^{1}H and 75.5 MHz for ^{13}C) and on a Bruker AC-500 spectrometer (500 MHz for ^{1}H and 125.7 MHz for ^{13}C). Chemical shifts are reported in parts per million (ppm) relative to an internal solvent reference. Recorded peaks are listed in the order multiplicity (s, singlet; d, doublet; dd, doublet of doublets; m, multiplet), coupling constants, and number of protons. Assignments of individual ^{13}C and ^{1}H resonances are supported by DEPT experiments and 2D correlation experiments (COSY, HSQCed or HMBC) when necessary. High resolution mass spectra (HRMS) were performed by the Mass Spectrometry General Service at the University of the Basque Country using an ultra-performance liquid chromatograph (Acquity UPLC, Waters Chromatography.), in tandem with a QTOF mass spectrometer (SYNAPT G2 HDMS, Waters Chromatography), with an electrospray ionization source in a positive mode.

Schotten-Baumann reaction. Synthesis of *N*-acyl riluzole derivatives **1a**-c. General procedure. To a solution of riluzole (1 mmol) in dry DMF (4 mL), the adequate anhydride (2.2 mmol) and *N, N*-diisopropylethylamine (DIPEA) (3 mmol) were added. The reaction mixture was stirred at 60°C for 24 h and, then, $CH_{2c}l_2$ (20 mL) was added. The organic phase washed with water (3 x 20 mL), dried over anhydrous Na_2SO_4, filtered and concentrated under vacuum. Purification by column chromatography (silica gel, petroleum ether/AcOEt) afforded the corresponding *N*-acylbenzothiazol-2-amines **1a**-c.

N-[6-(trifluoromethoxy) benzo[*d*]thiazol-2-yl) benzamide (**1a**). According to General Procedure, riluzole (103.3 mg, 0.47 mmol) in dry DMF (4 mL) was treated with benzoic anhydride (257.1 mg, 1.02 mmol) and DIPEA (0.24 mL, 1.40 mmol). After work-up, purification by column chromatography (silica gel, petroleum ether/AcOEt 7:3) provided **1a** (140.1 mg, 89 %) as a white solid: m. p. (petroleum ether/ AcOEt): 180-181°C. IR (ATR): 3144, 3056, 2963, 1679, 1610, 1542, 1460 cm^{-1}. ^{1}H NMR (300 MHz, $CDCl_3$): δ 6.99 (d, *J* = 8.9 Hz, 1H), 7.06 (dd, *J* = 8.9, 1.2 Hz, 1H), 7.39 (t, *J* = 7.5 Hz, 2H), 7.56 (t, *J* = 7.5 Hz, 1H), 7.70 (d, J = 1.2 ,1H), 8.00 (d, *J* = 7.5 Hz, 2H), 12.38 (broad s, 1H). $^{13}C\{^{1}H\}$ NMR (75.5 MHz, $CDCl_3$): δ 114.1, 119.9, 120.5 (q, *J* = 257.2 Hz), 121.3, 128.1, 129.1, 131.9, 132.7, 133.3, 145.4, 146.2, 161.1, 166.4. MS (ESI) *m/z* (rel intensity): 339 (MH^+, 100). HRMS (ESI-TOF): calcd for $C_{15}H_{10}F_3N_2O_2S$ [MH^+]: 339.0415; found, 339.0420.

N-[6-(trifluoromethoxy) benzo[*d*]thiazol-2-yl) butyramide (**1b**). According to General Procedure, riluzole (107.4 mg, 0.48 mmol) in dry DMF (4 mL) was treated with butyric anhydride (168.2 mg, 1.06 mmol) and DIPEA (0.25 mL, 1.45 mmol). After work-up, purification by column chromatography (silica gel, petroleum ether/AcOEt 9:1) provided **1b** (115.8 mg, 79 %) as a brown solid: m. p. (petroleum ether/ AcOEt): 167-168°C. IR (ATR): 3434, 3060, 1714, 1633, 1531, 1443 cm^{-1}. ^{1}H NMR (300 MHz, $CDCl_3$): δ 1.00-1.07 (m, 3H), 1.70-1.88 (m, 2H), 2.39-2.57 (m, 2H), 7.30-7.33 (m, 1H), 7.67-7.70 (m, 2H), 11.89 (broad s, 1H). $^{13}C\{^{1}H\}$ NMR (75.5 MHz, $CDCl_3$): δ 13.6 (CH_3, major rotamer), 13.7 (CH_3, minor rotamer) 18.3 ($CH_{2c}H_{2c}H_3$, major rotamer), 18.5 ($CH_{2c}H_{2c}H_3$, minor rotamer), 36.5 ($\underline{C}H_{2c}H_{2c}H_3$, minor rotamer), 38.1 ($\underline{C}H_{2c}H_{2c}H_3$, major rotamer), 114.4, 120.4, 120.5, 120.5 (q, *J* = 257.4 Hz),

132.0, 145.4, 161.1, 172.2, 179.7. MS (ESI) *m/z* (rel intensity): 305 (MH^+, 100). HRMS (ESI-TOF): calcd for $C_{12}H_{12}F_3N_2O_2S$ [MH^+]: 305.0572; found, 305.0576.

N-[6-(trifluoromethoxy) benzo[*d*]thiazol-2-yl) isobutyramide (**1c**). According to General Procedure, riluzole (107.5 mg, 0.48 mmol) in dry DMF (4 mL) was treated with isobutyric anhydride (168.4 mg, 1.06 mmol) and DIPEA (0.25 mL, 1.45 mmol). After work-up, purification by column chromatography (silica gel, petroleum ether/AcOEt 7:3) provided **1c** (107.5 mg, 73 %) as a white solid: m. p. (petroleum ether/ AcOEt): 134-135°C. IR (ATR): 3247, 3060, 2974, 1697, 1608, 1545, 1457 cm^{-1}. ^{1}H NMR (300 MHz, $CDCl_3$): δ 1.28 (d, *J* = 6.0 Hz, 6H), 2.61-2.75 (m, 1H), 7.32-7.35 (m, 1H, 1H), 7.72-7.75 (m, 2H), 11.09 (broad s, 1H). $^{13}C\{^1H\}$ NMR (75.5 MHz, $CDCl_3$): 19.0 (minor rotamer), 19.1 (major rotamer), 34.2 (minor rotamer), 35.5 (major rotamer), 114.5, 120.2, 120.6 (q, *J* = 257.2 Hz), 120.9, 132.9, 145.5, 146.5, 160.5, 176.0. MS (ESI) *m/z* (rel intensity): 305 (MH^+, 100), 235 (23), 306 (10). HRMS (ESI-TOF): calcd for $C_{12}H_{12}F_3N_2O_2S$ [MH^+]: 305.0572; found, 305.0575.

Synthesis of *N*-[6-(trifluoromethoxy) benzo[*d*]thiazol-2-yl) phenylacetamide (**1d**). To a solution of riluzole (101.9 mg, 0.46 mmol) in dry $CH_{2c}l_2$ (4 mL), benzoic acid (62.0 mg, 0.46 mmol) and dimetylaminopyridine (DMAP) (1.4 mg, 0.01 mmol) were added under argon atmosphere. Then *N, N'*-dicyclohexylcarbodiimide (DCC) (94.6 mg, 0.46 mmol) was added in portions and the reaction mixture was stirred at room temperature for 24 h. Then, $CH_{2c}l_2$ (15 mL) was added and the organic phase washed with an aqueous solution of 10% HCl (10 mL) and a saturated aqueous solution of $NaHCO_3$ (10 mL). The combined organic extracts were dried over anhydrous Na_2SO_4, filtered and concentrated under vacuum. Purification by column chromatography (silica gel, petroleum ether/AcOEt 6:4) afforded *N*-benzoylbenzothiazol-2-

amine **1d** (133.4 mg, 87 %) as a white solid: m. p. (petroleum ether/AcOEt): 157-158°C. IR (ATR): 3227, 3034, 2973, 1690, 1605, 1549, 1499 cm^{-1}. ^{1}H NMR (300 MHz, $CDCl_3$): δ 3.87 (s, 2H), 7.20-7.22 (m, 2H), 7.30-7.32 (m, 4H), 7.70 -7.73 (m, 2H,), 10.64 (broad s, 1H). $^{13}C\{^{1}H\}$ NMR (75.5 MHz, $CDCl_3$): δ 43.3, 114.4, 120.3, 120.6 (q, *J* = 257.3 Hz), 121.3, 128.0, 129.2, 129.3, 132.6, 133.0, 145.5 (q, *J* = 2.1 Hz), 146.6, 159.7, 169.9. MS (ESI) *m/z* (rel intensity): 353 (MH^+, 100). HRMS (ESI-TOF): calcd for $C_{16}H_{12}F_3N_2O_2S$ [MH^+]: 353.0572; found, 353.0577.

Synthesis of brominated benzo [*d*]thiazol-2-amines **2a**-e. General procedure. KSCN (4 mmol) and a solution of Br_2 (1 mmol) in HAcO (2 mL) were added to a solution of the corresponding bromoaniline (1 mmol) in HAcO (7 mL) at room temperature and the mixture was stirred for 16h. The reaction was quenched with saturated $NaHCO_3$ (150 mL) and the aqueous phase extracted with AcOEt (2 x 50 mL). The combined organic extracts were washed with saturated NaCl (2 x 50 mL), dried over anhydrous Na_2SO_4 and concentrated *in vacuo*. Purification by column chromatography (silica gel, petroleum ether/AcOEt 7:3) afforded **2a**-c in moderate yields. All data are in agreement with literature.[4]

^{1}H and ^{13}C-NMR spectra for compounds **1a**-d

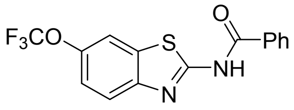

1a

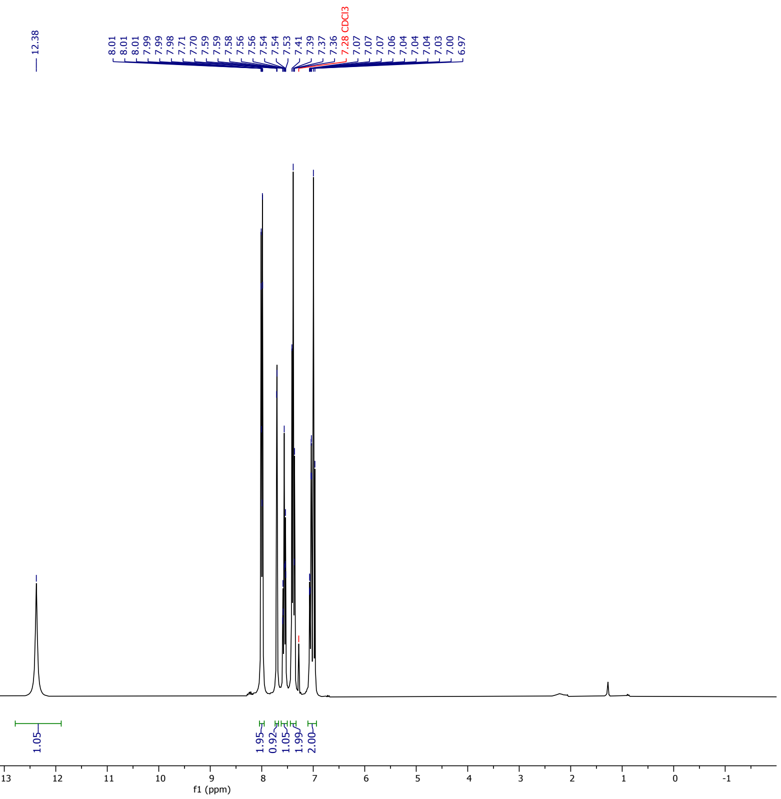

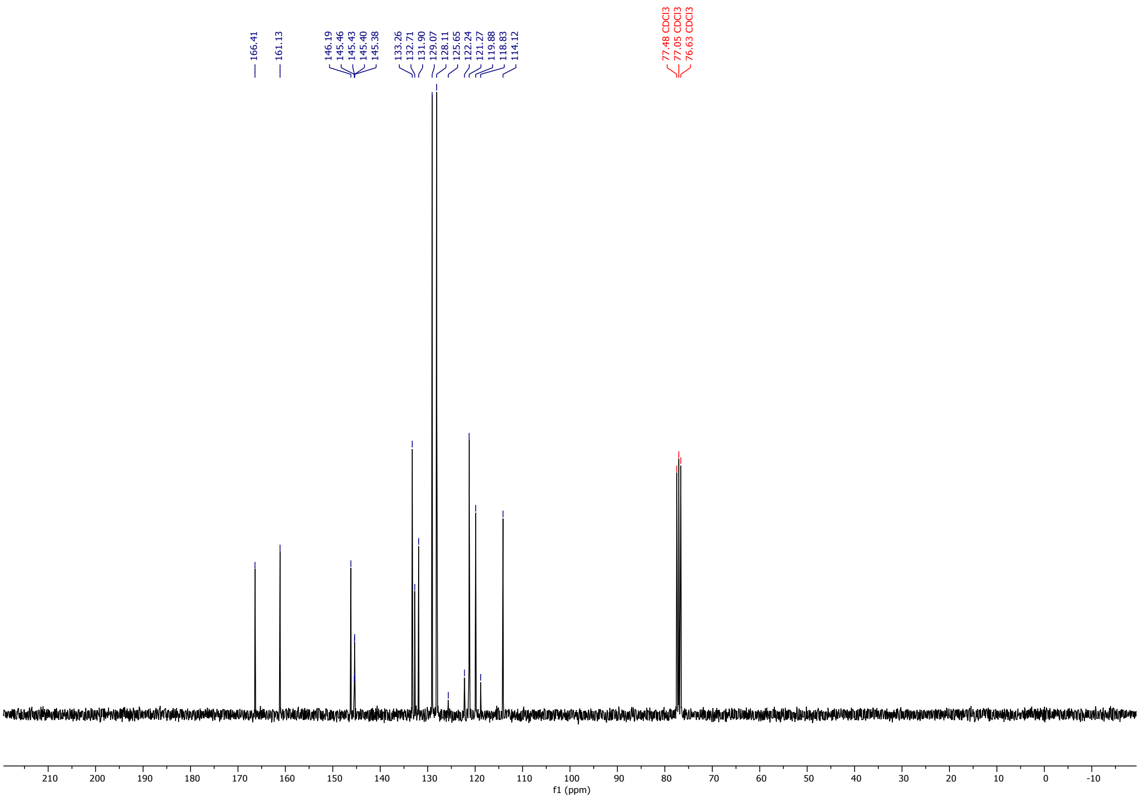

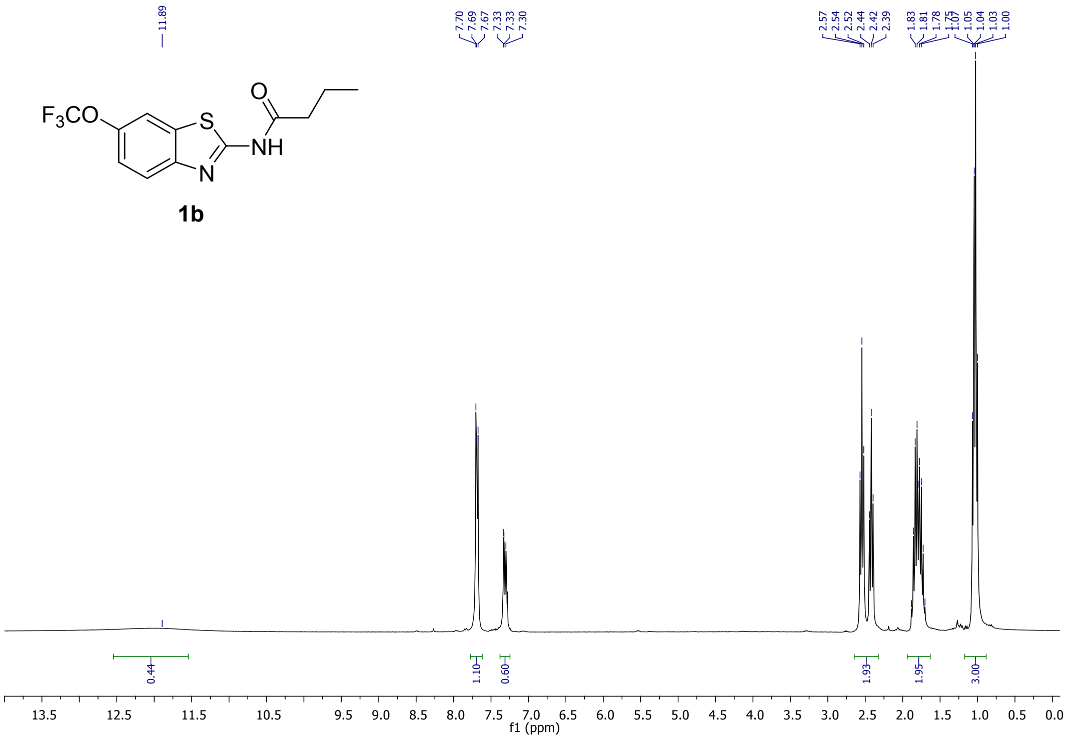
F3CO
S
N
NH
O
1b
11.89
7.70
7.69
7.67
7.33
7.33
7.30
2.57
2.54
2.52
2.44
2.42
2.39
1.83
1.81
1.78
1.75
1.07
1.05
1.04
1.03
1.00
0.44
1.10
0.60
1.93
1.95
3.00
f1 (ppm)

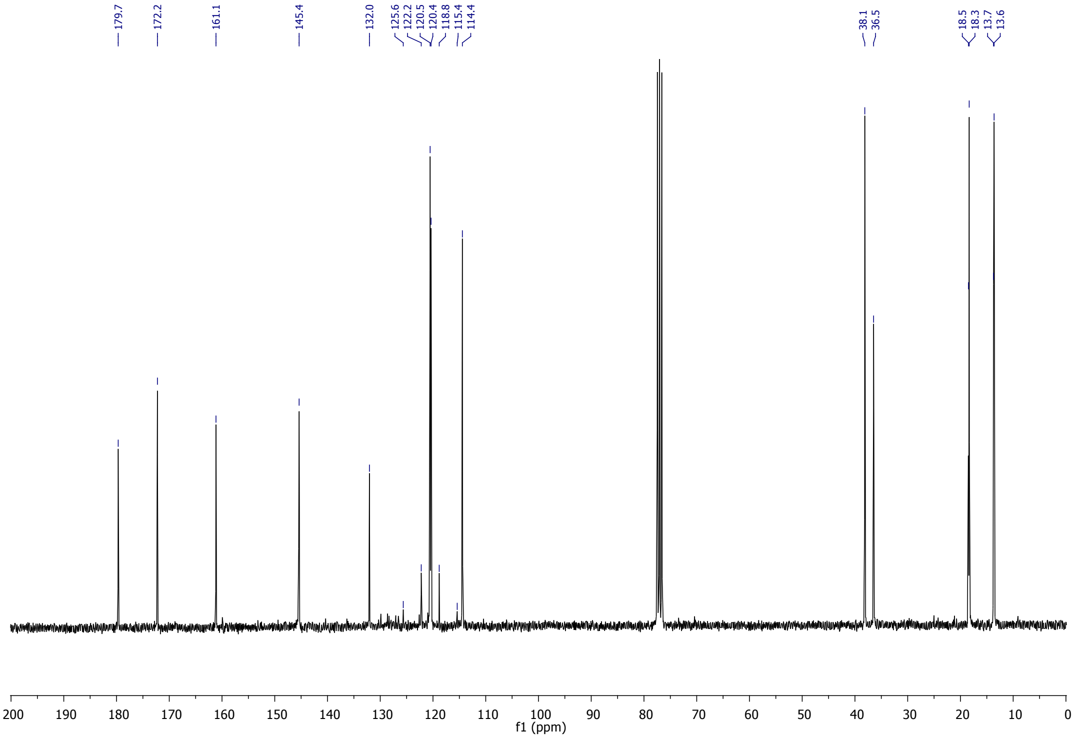
179.7
172.2
161.1
145.4
132.0
125.6
122.2
120.5
120.4
118.8
115.4
114.4
38.1
36.5
18.5
18.3
13.7
13.6
f1 (ppm)

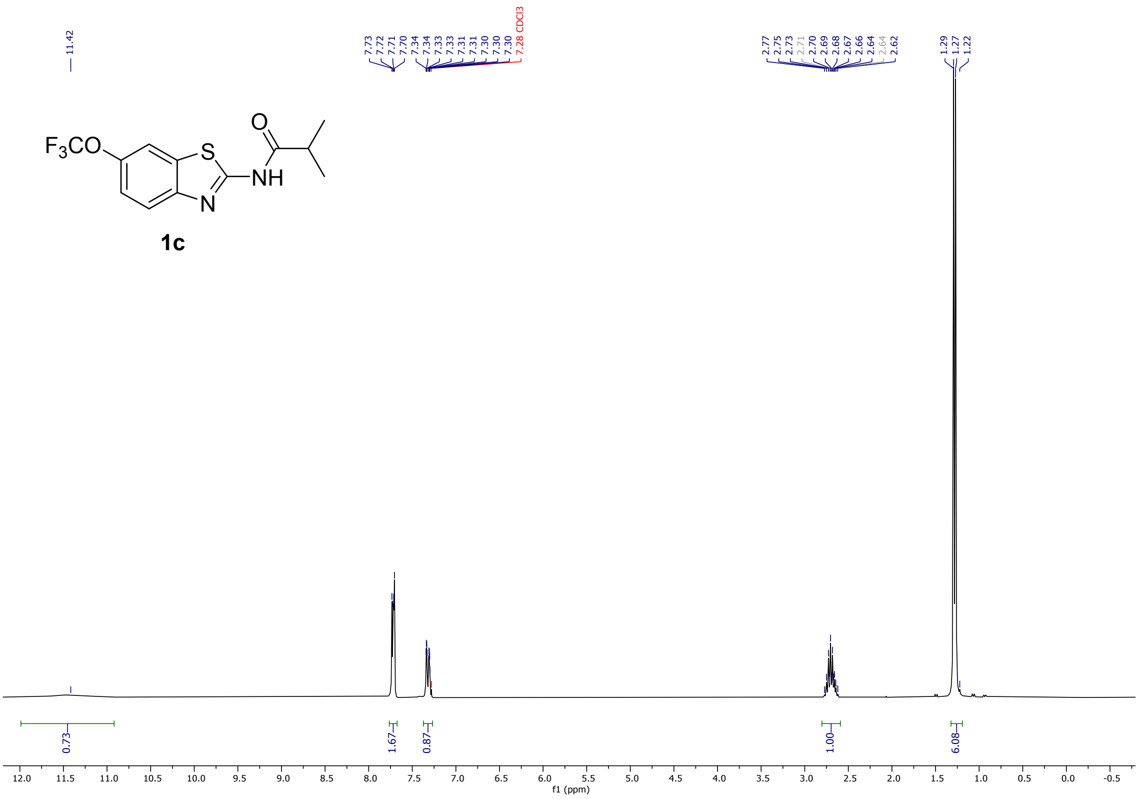
F3CO
S
N
NH
O
1c
11.42
7.73 7.72 7.71 7.70 7.34 7.34 7.33 7.33 7.31 7.31 7.30 7.30 7.30
7.28 CDCl3
2.77 2.75 2.73 2.71 2.70 2.69 2.68 2.67 2.66 2.64 2.62
1.29 1.27 1.22
0.73
1.67
0.87
1.00
6.08
12.0 11.5 11.0 10.5 10.0 9.5 9.0 8.5 8.0 7.5 7.0 6.5 6.0 5.5 5.0 4.5 4.0 3.5 3.0 2.5 2.0 1.5 1.0 0.5 0.0 -0.5
f1 (ppm)

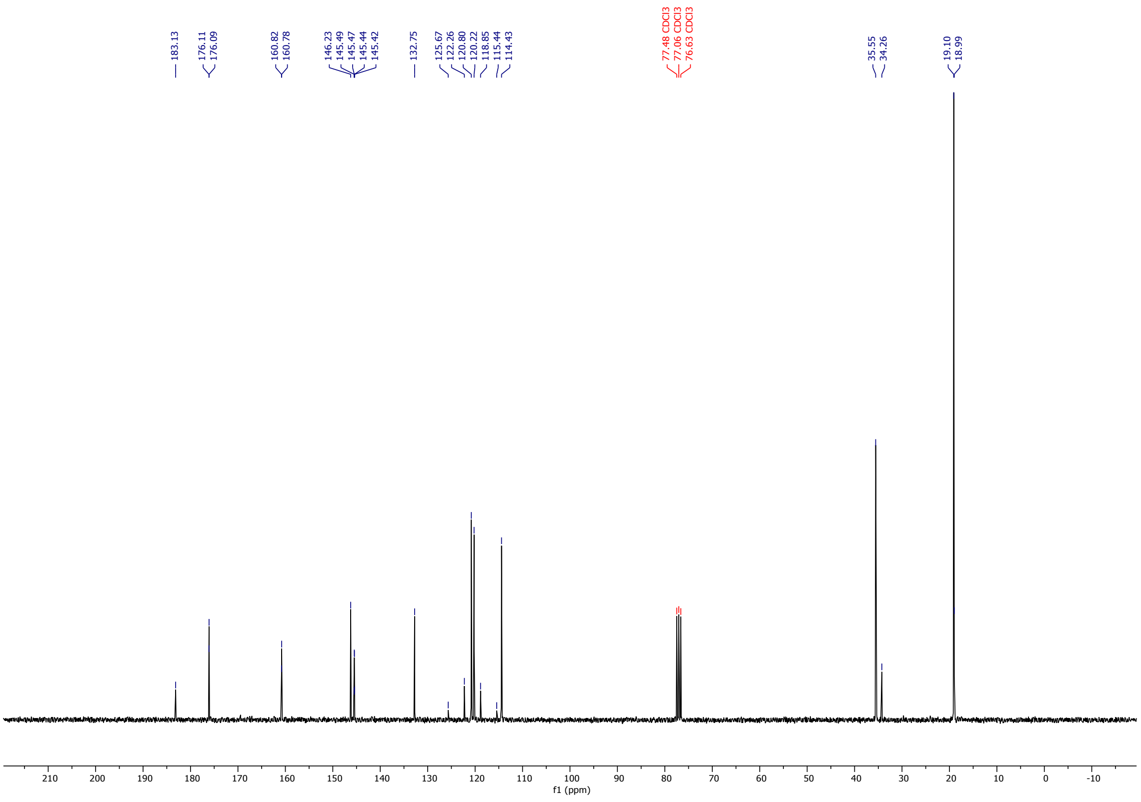
183.13
176.11 176.09
160.82 160.78
146.23 145.49 145.47 145.44 145.42
132.75
125.67 122.26 120.80 120.22 118.85 115.44 114.43
77.48 CDCl3
77.06 CDCl3
76.63 CDCl3
35.55 34.26
19.10 18.99
210 200 190 180 170 160 150 140 130 120 110 100 90 80 70 60 50 40 30 20 10 0 -10
f1 (ppm)

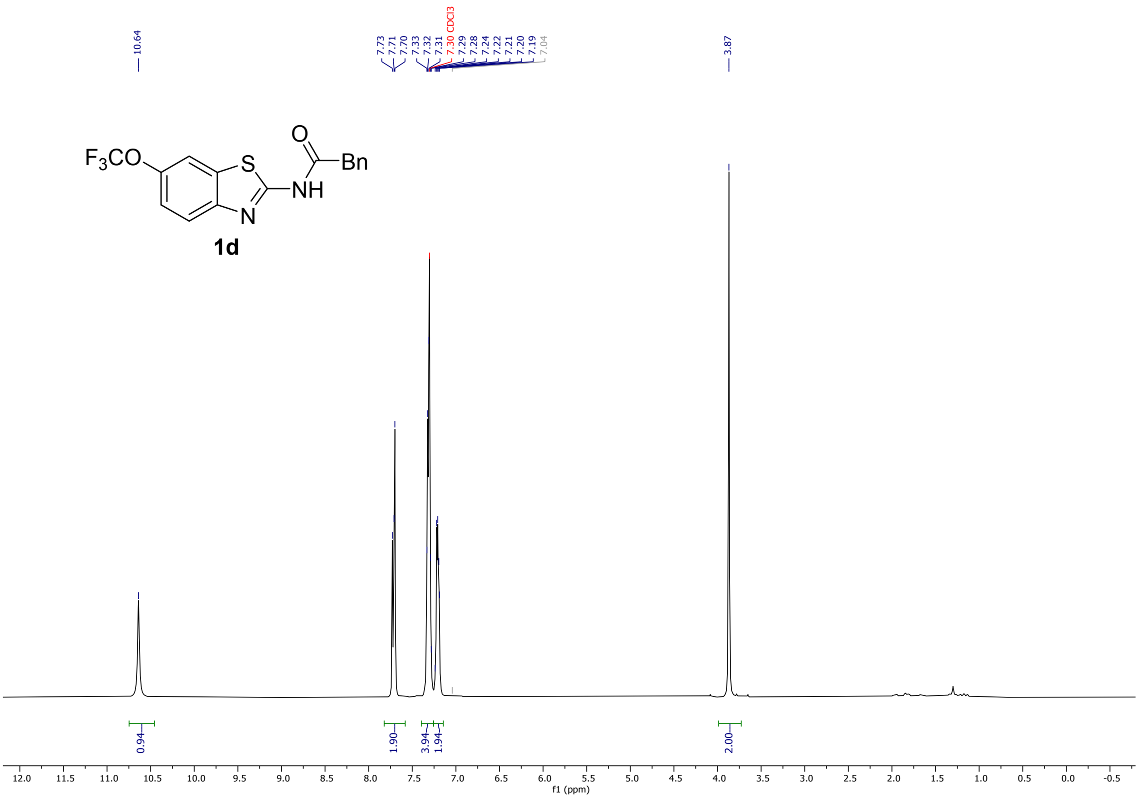

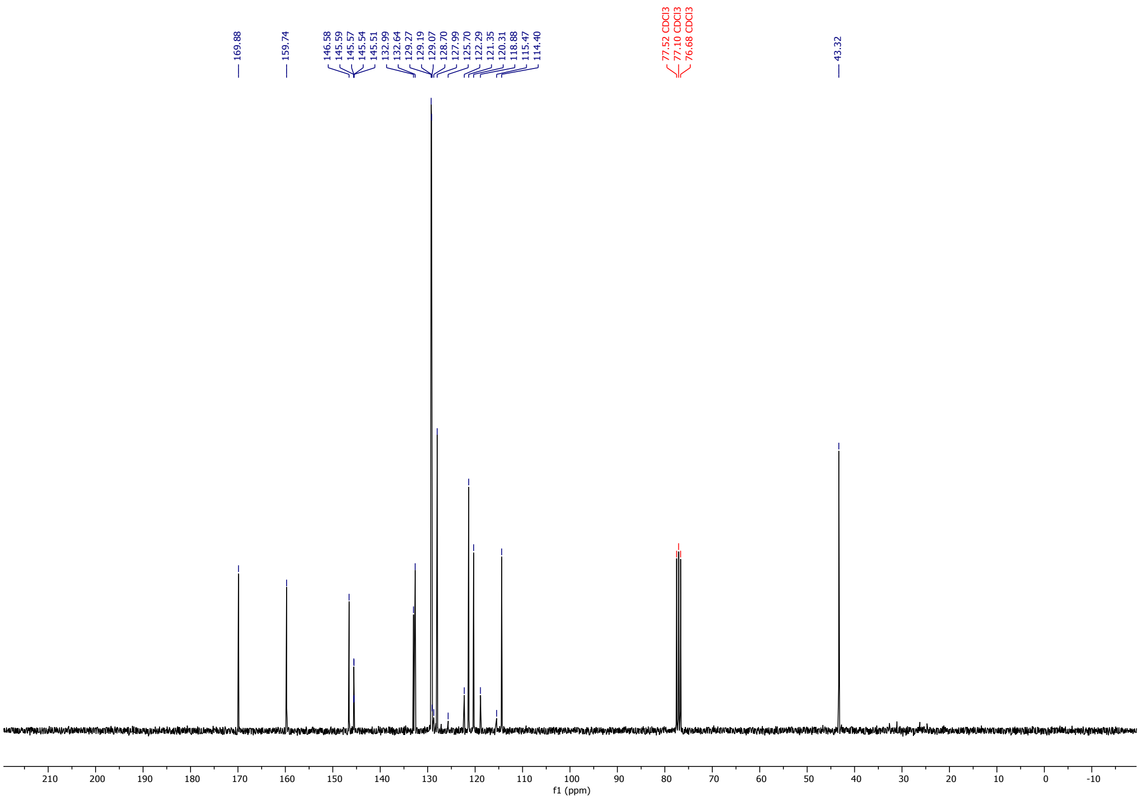

7. Biological assay of riluzole derivatives additional details

Monitoring Ca^{2+} dependent interactions between CaM and targets can be achieved through biosensors such as those that consist of CaM fused to the target protein and two fluorophores, a donor and acceptor at the N- and C-termini, that enable tracking of interactions. The YC-Nano15 calcium biosensor,[5] serving as a template, was modified by replacing the M13 sequence with the sequence of the S4S5 linker from the SK4 channel sequence. The modified construct was cloned into a pProEX-HTc plasmid (Invitrogen) with a 6xHis tag at the N-terminus and transformed into BL21 (DE3) cells (Novagen) via electroporation.

Cells were grown at 37 ˚C in 1 L of LB medium with ampicillin until an A_{600} between 0.6-0.8 was achieved. Fusion protein expression was induced with 0.5 mM IPTG overnight at 20 ˚C. Cells were harvested by centrifugation (9,000 g for 9 min) and re-suspended in 40 ml Buffer A (KCl 120 mM, K-HEPES 50 mM [pH 7.4], NaCl 5 mM, DTT 500 μM, PMSF 1 mM, protease inhibitor EDTA free: Roche, Ref. 04693132001). After sonication-based lysis (15 s ON, 15 s OFF, 25 cycles, 7.5 μm), the slurry underwent centrifugation (30,000 g for 30 min), with the supernatant filtered (0.20 μm) and transferred to a clean tube.

Affinity purification using a His-Trap-talon column, equilibrated with fluorescence buffer (KCl 120 mM, Hepes 50 mM, NaCl 5 mM, EGTA 5 mM), was performed on the supernatant. Size-exclusion chromatography utilized a Superdex 200 pg 26/60 column (GE Healthcare, ref. 28–9893) pre-equilibrated with fluorescence buffer. The soluble monomeric protein-containing fractions were dialyzed overnight against fluorescence buffer, followed by centrifugation at 14,000 g for 10 min to eliminate aggregates.

For drug titration, 10 mM drug solutions in DMSO were added at a final concentration of 100 μM with 1% DMSO. At this concentration, DMSO had negligible effects on the biosensors' spectra emission. FRET measurements were conducted in the absence and presence of free Ca^{2+} concentrations (between 0 and 1,600 nM). Fura-2 was employed to estimate free Ca^{2+} concentrations following the manufacturer's instructions (Invitrogen).

Förster resonance energy transfer (FRET) changes were monitored using a Fluoromax-3 fluorimeter. Emission spectra were collected post-excitation at 435 nm (maximal absorbance wavelength for the donor CFP) from 450 to 570 nm. The FRET index was computed as the emission ratio at 475 nm and 525 nm, corresponding to the peak emission wavelengths for the donor (CFP) and acceptor (YFP) fluorescent proteins, respectively.

8. Docking study additional details

The predicted binding poses were analyzed with binding pose metadynamics. Briefly, 10 metadynamics trajectories of 10 ns each were performed for every ligand, with the RMSD with respect to the docked ligand conformation as the collective variable, and statistics were collected during the last 2 ns of the simulation. The stability of predicted conformations was measured in terms of two variables: the mean RMSD with respect to the docked conformation during the last 2 ns of the simulation (PoseScore), and the percentage of contacts that are preserved during the last 2 ns of the simulation (ContactScore), which we define as a pair of atoms from the ligand and the receptor within 3 A in the docked structure. The CompScore combines both of these scores to give a final estimate of the stability of the conformations as follows:

$$CompScore = PoseScore - 5 * ContactScore \qquad (1)$$

The starting structures for MD simulation were prepared using the CHARMM-GUI[6] input generator. The bound structures were solvated in a box of TIP3P water molecules and neutralized with K^+ and Clions. For the parameterization of the protein and the ligands, the FF14SB[7] and GAFF2[8] force fields were used, respectively. The system was then minimized and equilibrated for 500 ps in the NVT ensemble using the OpenMM[9] simulation engine. During equilibration, protein and ligand heavy atoms were restrained using a harmonic potential with a force constant of 5 kcal/mol.

The production simulations were carried out on the NVT ensemble, using a Langevin integrator with a temperature of 300 K, a friction coefficient of 1 ps^{-1} and a timestep of 4 fs. To conduct metadynamics, Gaussian potentials with hill height and width of 0.3 kcal/mol and 0.02 Å, respectively, were used.

9. IFPTML predictive study additional details

To be able to carry out this prediction, it was necessary to create a new dataset with preclinical assays where the main protein tested was CaM or related proteins. In fact, the followed methodology resembles the one to create the model. Firstly, the preclinical assay's data used was the same as the data used to develop the IFPTML model; this included different preclinical assays tested on proteins related to Ca^{2+} signaling pathway linked to CaM. Besides, to further clean the dataset, all assays that were duplicated were deleted taking into account the following assay conditions (c_j): target name (c_1), assay cell type (c_2), assay tissue name (c_3), target organism (c_4), assay organism (c_5), target type (c_6), assay subcellular fraction (c_7), buffer (c_8), standard relation (c_9) and assay type (c_{10}). Moreover, the information related to riluzole and its derivatives was added. To do so, the data was duplicated 8 times (one for each proposed drug).

At this point 1648 unique assays were left. Furthermore, the molecular descriptors of the 8 molecules were searched using MARCH-INSIDE 2.0® (*Markovian Chemicals in Silico Design*) program.[10–13] The MARCH-INSIDE needed molecular descriptors included the ones used on the resulting model.

Once the descriptors of the data series were collected, the Perturbation Theory Operators[14] were calculated. In fact, the PT operators or MA, $\langle D_i(c_j) \rangle$, that were used to calculate the delta of each case were the same ones from the dataset used to achieve the model. That way, the perturbation theory operators were also divided into two different sections based on the conditions, i.e. $c_{assay} = (c_1, c_2, c_3, c_4, c_5)$ and $c_{dat} = (c_6, c_7, c_8, c_9, c_{10})$ and the deltas were calculated following Equation 1 for each case.

As soon as all the data was gathered, predictions were generated using the IFPTML-XGB model, which was computed in Jupyter Notebook. With these predictions, the different scoring functions v_{ij} of the drugs (riluzole, **1a**-**2c**) were calculated. To enable comparison with the results for riluzole, the relative value $\Delta f(v_{ij})_{calc}$ (%) (Equation 5) was calculated by substituting the outputs of the LDA model for the derivative $f(v_{ij})_{calc}$ and riluzole $f(v_{riluzolej})_{calc}$ into the following equation, only taking into account the binding assay types among all the data.

$$\Delta f(v_{ij})_{calc}(\%) = 100 \cdot \frac{\left[f(v_{ij})_{calc} - f(v_{riluzolej})_{calc}\right]}{f(v_{riluzolej})_{calc}} \qquad (2)$$

Supporting Information I - References

(1) Pedregosa, F.; Varoquaux, G.; Gramfort, A.; Michel, V.; Thirion, B.; Grisel, O.; Blondel, M.; Prettenhofer, P.; Weiss, R.; Dubourg, V.; Vanderplas, J.; Passos, A.; Cournapeau, D.; Brucher, M.; Perrot, M.; Duchesnay, É. Scikit-Learn: Machine Learning in Python. *Journal of Machine Learning Research* **2011**, *12*, 2825–2830.

(2) Lukauskis, D.; Samways, M. L.; Aureli, S.; Cossins, B. P.; Taylor, R. D.; Gervasio, F. L. Open Binding Pose Metadynamics: An Effective Approach for the Ranking of Protein–Ligand Binding Poses. *Journal of Chemical Information and Modeling* **2022**, *62* (23), 6209–6216. https://doi.org/10.1021/acs.jcim.2c01142.

(3) Kumari, R.; Kumar, R.; Open Source Drug Discovery Consortium; Lynn, A. *G_mmpbsa* —A GROMACS Tool for High-Throughput MM-PBSA Calculations. *Journal of Chemical Information and Modeling* **2014**, *54* (7), 1951–1962. https://doi.org/10.1021/ci500020m.

(4) Piscitelli, F.; Ballatore, C.; Smith, A. B. Solid Phase Synthesis of 2-Aminobenzothiazoles. *Bioorganic & Medicinal Chemistry Letters* **2010**, *20* (2), 644–648. https://doi.org/10.1016/j.bmcl.2009.11.055.

(5) Horikawa, K.; Yamada, Y.; Matsuda, T.; Kobayashi, K.; Hashimoto, M.; Matsu-ura, T.; Miyawaki, A.; Michikawa, T.; Mikoshiba, K.; Nagai, T. Spontaneous Network Activity Visualized by Ultrasensitive Ca2+ Indicators, Yellow Cameleon-Nano. *Nature Methods* **2010**, *7* (9), 729–732. https://doi.org/10.1038/nmeth.1488.

(6) Jo, S.; Kim, T.; Iyer, V. G.; Im, W. CHARMM-GUI: A Web-Based Graphical User Interface for CHARMM. *Journal of Computational Chemistry* **2008**, *29* (11), 1859–1865. https://doi.org/10.1002/jcc.20945.

(7) Lindorff-Larsen, K.; Piana, S.; Palmo, K.; Maragakis, P.; Klepeis, J. L.; Dror, R. O.; Shaw, D. E. Improved Side-Chain Torsion Potentials for the Amber ff99SB Protein Force Field: Improved Protein Side-Chain Potentials. *Proteins* **2010**, *78* (8), 1950–1958. https://doi.org/10.1002/prot.22711.

(8) Wang, J.; Wolf, R. M.; Caldwell, J. W.; Kollman, P. A.; Case, D. A. Development and Testing of a General Amber Force Field. *Journal of Computational Chemistry* **2004**, *25* (9), 1157–1174. https://doi.org/10.1002/jcc.20035.

(9) Eastman, P.; Swails, J.; Chodera, J. D.; McGibbon, R. T.; Zhao, Y.; Beauchamp, K. A.; Wang, L.-P.; Simmonett, A. C.; Harrigan, M. P.; Stern, C. D.; Wiewiora, R. P.; Brooks, B. R.; Pande, V. S. OpenMM 7: Rapid Development of High Performance Algorithms for Molecular Dynamics. *PLOS Computational Biology* **2017**, *13* (7), e1005659. https://doi.org/10.1371/journal.pcbi.1005659.

(10) González Díaz, H.; Olazabal, E.; Castañedo, N.; Sánchez, I. H.; Morales, A.; Serrano, H. S.; González, J.; De Armas, R. R. Markovian Chemicals “in Silico” Design (MARCH-INSIDE), a Promising Approach for Computer Aided Molecular Design II: Experimental and Theoretical Assessment of a Novel Method for Virtual Screening of Fasciolicides. *Journal of Molecular Modeling* **2002**, *8* (8), 237–245. https://doi.org/10.1007/s00894-002-0088-7.

(11) Gonzáles-Díaz, H.; Gia, O.; Uriarte, E.; Hernádez, I.; Ramos, R.; Chaviano, M.; Seijo, S.; Castillo, J. A.; Morales, L.; Santana, L.; Akpaloo, D.; Molina, E.; Cruz, M.; Torres, L. A.; Cabrera, M. A. Markovian Chemicals "in Silico" Design (MARCH-INSIDE), a Promising Approach for Computer-Aided Molecular Design I: Discovery of Anticancer Compounds. . *Journal of Molecular Modeling* **2003**, *9* (6), 395–407. https://doi.org/10.1007/s00894-003-0148-7.

(12) González-Díaz, H.; Torres-Gómez, L. A.; Guevara, Y.; Almeida, M. S.; Molina, R.; Castañedo, N.; Santana, L.; Uriarte, E. Markovian Chemicals "in Silico" Design (MARCH-INSIDE), a Promising Approach for Computer-Aided Molecular Design III: 2.5D Indices for the Discovery of Antibacterials. *Journal of Molecular Modeling* **2005**, *11* (2), 116–123. https://doi.org/10.1007/s00894-004-0228-3.

(13) Gonzalez-Diaz, H.; Duardo-Sanchez, A.; M. Ubeira, F.; Prado-Prado, F.; G. Perez-Montoto, L.; Concu, R.; Podda, G.; Shen, B. Review of MARCH-INSIDE & Complex Networks Prediction of Drugs: ADMET, Anti-Parasite Activity, Metabolizing Enzymes and Cardiotoxicity Proteome Biomarkers. *Current Drug Metabolism* **2010**, *11* (4), 379–406. https://doi.org/10.2174/138920010791514225.

(14) González-Díaz, H.; Arrasate, S.; Gomez-SanJuan, A.; Sotomayor, N.; Lete, E.; Besada-Porto, L.; M. Ruso, J. General Theory for Multiple Input-Output Perturbations in Complex Molecular Systems. 1. Linear QSPR Electronegativity Models in Physical, Organic, and Medicinal Chemistry. *Current Topics in Medicinal Chemistry* **2013**, *13* (14), 1713–1741.
